# Clinical Trial to Evaluate the Safety and Immunogenicity of a Trivalent Surface Antigen Seasonal Influenza Vaccine Produced in Mammalian Cell Culture and Administered to Young and Elderly Adults with and without A(H1N1) Pre-Vaccination

**DOI:** 10.1371/journal.pone.0070866

**Published:** 2013-08-16

**Authors:** Micha Loebermann, Ulrich Voss, Seetha Meyer, Dietrich Bosse, Carlos Fritzsche, Sebastian Klammt, Silvius Frimmel, Diana Riebold, Emil C. Reisinger

**Affiliations:** 1 Department of Tropical Medicine, Infectious Diseases and Nephrology, University of Rostock, Rostock, Germany; 2 Novartis Vaccines and Diagnostics GmbH, Marburg, Germany; Glaxo Smith Kline, Denmark

## Abstract

Vaccination against influenza is an important means of reducing morbidity and mortality in subjects at risk. The prevalent viral strains responsible for seasonal epidemics usually change annually, but the WHO recommendations for the 2011/2012-season in the Northern hemisphere included the same antigens as for the previous season.

We conducted a single-center, single-arm study involving 62 younger (18–60 years) and 64 older (>60 years) adults to test the immunogenicity, safety and tolerability of a trivalent surface antigen, inactivated influenza vaccine produced in mammalian cell-culture. The vaccine contained 15 µg hemagglutinin of each of the virus strains recommended for the 2011–2012 Northern hemisphere winter season (A/California/7/09 (H1N1)-; A/Perth/16/09 (H3N2)-; B/Brisbane/60/08-like strain) in a non-adjuvanted preservative-free formulation. Antibody response was measured by hemagglutination inhibition 21 days after immunization. Adverse events and safety were assessed using subject diary cards and telephone interviews.

Seroconversion or a 4-fold antibody increase in antibody titers was detectable against A(H1N1) in 68% of both younger and older adults, against A(H3N2) in 53% and 27%, and against the B influenza strain in 35% and 17%. Antibody titers of 40 or more were observed against A(H1N1) in 87% and 90% of younger and older adults, against A(H3N2) in 98% and 98%, and against the B influenza strain in 93% and 90%. Pre-vaccination antibody titers were protective against A(H1N1), A(H3N2) and B in 38%, 58% and 58%, respectively, of younger and in 43%, 88% and 70% of older adults. Among subjects with previous A(H1N1) vaccination only 48% of younger and 47% of older adults had protective A(H1N1) antibodies at inclusion. Adverse reactions were generally mild. The most frequently reported reactions were pain at the injection site, myalgia and fatigue.

The vaccine generated protective antibodies against all three viral strains and had an acceptable safety profile in both younger and older adults.

**Trial Registration:**

ClinicalTrials.gov NCT01422512

## Introduction

Influenza vaccination is widely recommended to elderly subjects and at-risk adults as a means of preventing influenza infections. Mortality rates attributed to influenza infections are difficult to obtain but estimates indicate that subjects above the age of 75 may die in 2.5 to 8.1% of cases of seasonal influenza in Great Britain [1]. The viral strain responsible for the 2009 A(H1N1) pandemic was found to still be in circulation the following year, causing 50% of influenza cases in the 2010/11 influenza season in Europe [2]. Since the virus strains responsible for influenza vary from one winter to the other, the WHO closely monitors the spread of influenza worldwide and annually recommends the antigens to be used for seasonal influenza vaccines.

On the basis of these recommendations, influenza vaccines are produced annually and distributed worldwide to control the variants most likely to be causing the seasonal epidemic. Unlike other influenza vaccines, the inactivated mammalian cell culture-derived trivalent influenza vaccine Optaflu® no longer relies on a supply of embryonated eggs as a substrate for virus growth. Cell-derived vaccines can thus be produced more flexibly and variably and do not contain egg protein, a possible risk to those with egg allergies. Previous data have shown this subunit influenza vaccine to be safe, well tolerated and immunogenic [3]–[5]. It also met the criteria established for influenza vaccines by the Committee for Medicinal Products for Human Use (CHMP) [6] and by the FDA's Center for Biologics Evaluation and Research (CBER) [7].

The main aim of this study was to evaluate safety, clinical tolerability and immunogenicity in compliance with current EU guidelines [6] on the annual licensing of influenza vaccines in adult and elderly subjects. The Optaflu® formulation 2011–2012 contained the three strains A/California/7/2009 (H1N1)-like virus, A/Perth/16/2009 (H3N2)-like virus and B/Brisbane/60/2008-like virus as recommended by the WHO for the 2011/12 northern hemisphere influenza season [8]. Interestingly, the prevalent viral strains hadn't changed from the previous season, so the WHO recommendations for the 2010/11 and 2011/12 seasonal influenza vaccine in the Northern hemisphere included the same viral antigens [9].

## Methods

### Ethical Statement

The study protocol was approved by institutional review boards at each of the participating sites: “Ethikkommission an der Medizinischen Fakultät der Universität Rostock” and “Ethikkommission der Ärztekammer Hamburg”. All study participants provided written informed consent prior to participation. The study was performed in accordance with the Good Clinical Practice and current International Conference on Harmonization Guidelines. The trial is registered at Eudra CT, registration: 2010-024613-31 and ClinicalTrials.gov, registration: NCT01422512.

### Subjects and study procedures

The protocol for this trial and supporting CONSORT checklist are available as supporting information; see Checklist S1 (CONSORT checklist) and Protocol S1 (Trial protocol).

This phase III study was planned as a multicenter study. Eventually, however, it was possible to enroll the necessary number of subjects at one university-based center in Germany. The main study procedures were carried out as described in an earlier seasonal influenza vaccine study [10]. After providing informed consent, all subjects underwent a clinical examination and had their medical history taken. In females of child-bearing potential a urine pregnancy test was performed prior to immunization.

A total number of 126 healthy adults were enrolled in September 2011, six months after the end of 2010/2011 seasonal influenza activity. In this single-arm, open label study subjects were enrolled in two age groups, 62 in the non-elderly (18 to 60 year) subgroup, and 64 in the elderly subgroup, aged 61 years or above. All the subjects met the enrollment criteria listed in table 1. None of the subjects recalled having an influenza-like illness (ILI) during the preceding influenza season. Subjects received a single 0.5 ml dose of the vaccine which contained inactivated subunit antigens derived from influenza virus cultured in a mammalian cell line [4]. The preservative and adjuvant-free study vaccine was supplied in prefilled syringes and was administered in the deltoid muscle of the non-dominant arm.

**Table 1:** Main inclusion and exclusion criteria for the study population.

Inclusion criteria	Exclusion criteria
Informed consent	Behavioral or cognitive impairment or psychiatric disease that may interfere with the subject's ability to participate in the study
Aged 18 years and above	Any serious active disease (e.g. cancer; congestive heart failure; chronic obstructive pulmonary disease; autoimmune disease; hepatic disease; renal disease; diabetes mellitus type I; neurological or psychiatric disorders; asthma)
Health status compatible with vaccination	History of any anaphylactic reaction and/or serious allergic reaction following a vaccination, hypersensitivity to any component of the study vaccine
Ability to comply with all study requirements	Known or suspected impairment/alteration of immune function (excluding that normally associated with advanced age)
	Known or suspected history of drug or alcohol abuse
	Present or planned pregnancy; females of childbearing potential not willing to use acceptable measures of birth control
	Individuals not able to comprehend and to follow all required study procedures during the study
	History of any illness that might interfere with the study or pose an additional risk to the subject in the opinion of the investigator
	Confirmed seasonal or pandemic influenza infection within the last 6 months; or any seasonal or pandemic influenza vaccine within the last 6 months
	Any infection requiring systemic antibiotic or antiviral therapy in the last 7 days; fever (i.e. axillary temperature ≥38°C) within 3 days before study vaccination
	Participation in another clinical trial
	Any other vaccination within 4 weeks prior to enrollment and 4 weeks following study vaccine
	Reception of blood, blood products and/or plasma derivatives or immunoglobulin preparations within the past 12 weeks and during the study
	Body mass index above 35 kg/m^2^

### Safety and Immunogenicity

All the subjects were monitored at the study site for 30 minutes after the injection for immediate adverse reactions. Subjects recorded solicited and unsolicited local and systemic reactions in a standard study diary on the day of immunization and three days thereafter. Subjects were contacted by phone between 5 to 7 days after immunization to determine their clinical status and to ensure that data had been collected on their diary cards. The severity of adverse events (AEs) was graded as mild (no limitation of normal daily activities), moderate (some limitation of normal daily activities) and severe (unable to perform normal daily activities). The causal relationship between AEs and the vaccination was evaluated, with no relationship defined as follows: inconsistent timely relationship (too long an interval between the injection and the onset of the symptom or appearance of the symptom before the injection), or evidence that the symptom was definitely related to an etiology other than the study vaccine. All AEs that were possibly or probably related to the vaccine were defined as “related” to the vaccine. Solicited local (ecchymosis, erythema, induration, swelling, pain) and systemic (chills/shivering, malaise, myalgia, arthralgia, headache, sweating, fatigue, fever) reactions that occurred within 3 days of the day of vaccination were used as indicators of reactogenicity. As such, they were judged to be at least possibly related to the administration of the study vaccine. If a local or systemic reaction continued beyond day 4, it was additionally recorded as an AE.

Blood samples of approximately 10 ml were obtained prior to and 21 days (20 to 26 days) after vaccination. Sera were prepared within the hour and stored at −70°C until hemagglutination inhibition (HI) antibody titers were analyzed as previously reported [11] for each of the three antigens [A(H1N1), A(H3N2) and B] at Novartis Vaccines, Clinical Serology Laboratory, Marburg, Germany. For confirmation purposes, immunogenicity results for all the strains were also analyzed using the Single Radial Hemolysis (SRH) assay. In order to determine immunogenicity, the geometric mean titer (GMT) -or geometric mean area (GMA) for SRH- on day 1 and on day 22, the day 22/day 1 geometric mean ratios (GMR), the percentage of subjects achieving seroconversion or a significant increase in antibody titer and the percentage of subjects achieving an HI titer ≥40 (or an SRH area ≥25 mm^2^) on day 1 and on day 22 were ascertained. Seroconversion or a significant increase in antibody titer (negative pre-vaccination serum and a post-vaccination serum titer ≥40/area ≥25 mm^2^ or a four-fold or greater increase in titer from positive pre-vaccination titers) were used to evaluate the vaccine according to international guidelines [6].

### Statistical analysis

The study populations analyzed were a) all enrolled subjects; b) exposed subjects (those subjects enrolled in the study who actually received a study vaccination); c) an immunogenicity full analysis set (FAS) or modified intention-to-treat (MITT) population (i.e. all exposed subjects who provided at least one evaluable serum sample); d) a per protocol set (PPS) (i.e. all the subjects in the immunogenicity FAS who correctly received the vaccine, provided evaluable serum samples before and after vaccination and committed no major protocol violation); e) a safety set (all exposed subjects who provided post-vaccination safety data).

A sample size of 50 subjects per age group is required by the current CHMP guideline on the harmonization of requirements for influenza vaccines [6]. Assuming that the rate of non-evaluable subjects would be approximately 17.5%, the plan was to include a total of at least 63 subjects in each age group. A sample size calculation for direct comparison of the groups was not carried out prior to the initiation of the study since ethical considerations would not allow exceeding the numbers of subjects required by the CHMP.

All statistical analyses were performed using SAS® version 9.1 or higher (SAS Institute, Cary, NC). Where appropriate, the two-sided Fisher's exact test was used at a 95% confidence interval (CI) (this was based on a type 1 error probability (α) of 5%) to compare the number of subjects with local or systemic reactions after vaccination, and to compare subject characteristics between the two age groups. All reported p-values are two-sided; values of 0.05 or less were considered to indicate statistical significance. Safety data were summarized descriptively.

## Results

### Study subjects

**Figure 1:**
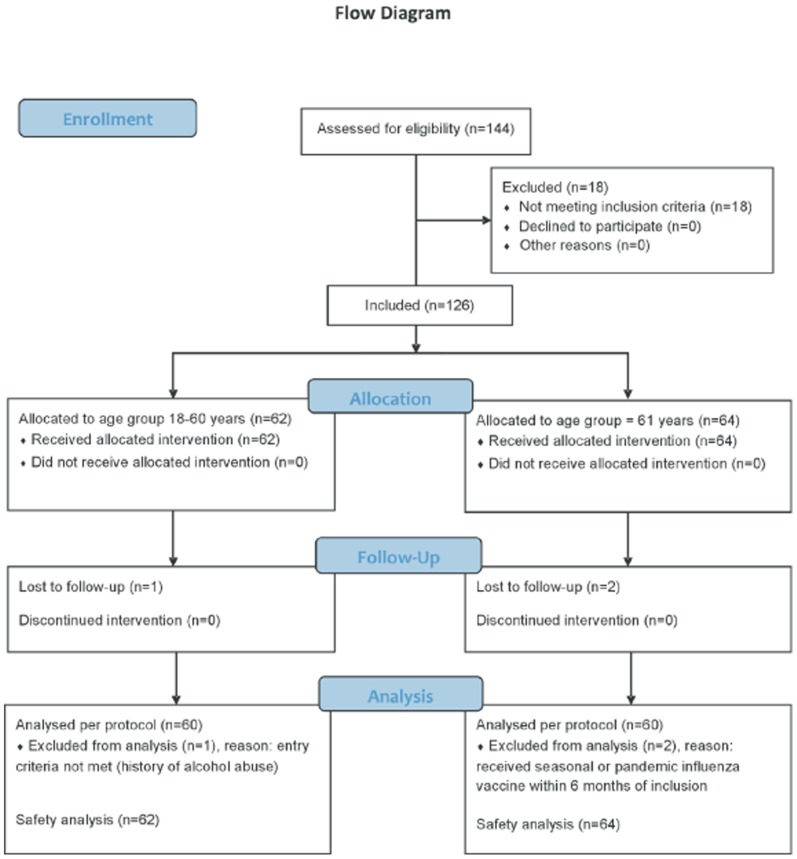
Flowchart of the number of subjects included and analyzed.

A total of 126 subjects were enrolled (62 aged 18–60 and 64 aged ≥61 years) and all 126 were included in the safety analysis (figure 1). The characteristics of the study population are summarized in table 2. Two non-elderly adults were erroneously enrolled in the elderly age group. The safety analysis, however, is based on the subjects' real age, i.e. 64 subjects aged 18–60 years and 62 aged ≥61 years. In all other analyses (demography, medical history, immunogenicity etc.) subjects were analyzed as they were enrolled (i.e. the 2 younger subjects were analyzed in the age group ≥61 years). Six subjects did not complete the study per protocol, 3 were lost to follow up and 3 met an exclusion criterion (history of alcohol or drug abuse and influenza vaccination in the 6 months prior to first visit).

**Table 2:** Demographic data of the study population.

	Number (%) of subjects			p
	18–60 YOA	≥61 YOA	TOTAL	
Enrolled	**62 (100%)**	**64 (100%)**	**126 (100%)**	
Completed protocol	60 (97%)	60 (94%)	120 (95%)	
Immunogenicity (FAS) analysis^1^	60 (97%)	60 (94%)	120 (95%)	
Immunogenicity (PP) analysis^1^	60 (97%)	60 (94%)	120 (95%)	
Exposed^2^	64 (100%)	62 (100%)	126 (98%)	
Safety analysis^2^	64 (100%)	62 (100%)	126 (98%)	
Premature withdrawals	2 (3%)	4 (6%)	6 (5%)	
Age (years):	39.7±12.0	68.5±6.0	54.3±17.3	<0.0001
Gender:				0.71
Male	27 (44%)	29 (45%)	56 (44%)	
Female	35 (56%)	35 (55%)	70 (56%)	
Females of child-bearing potential				
No	10/35 (29%)	35/35 (100%)	45/70 (64%)	
Yes	25/35 (71%)	0	25/70 (36%)	
Ethnic origin:				
Caucasian	62 (100%)	64 (100%)	126 (100%)	
Weight (kg):	75.56±12.58	77.58±14.70	76.59±13.68	0.73
Height (cm):	172.2±9.6	168.7±9.3	170.4±9.5	0.0147
Body Mass Index:	25.42±3.29	27.16±4.02	26.30±3.77	0.005
Previous seasonal vaccination				<0.0001
No	24 (39%)	6 (9%)	30 (24%)	
Yes	38 (61%)	56 (91%)	96 (78%)	
Previous pandemic vaccination				0.15
No	54 (87%)	58 (91%)	112 (89%)	
Yes	8 (13%)	5 (8%)	13 (10%)	
Met entry criteria				
Yes	61 (98%)	62 (97%)	123 (98%)	
No	1 (2%)	2 (3%)	3 (2%)	

YOA = years of age; categorical parameters: N(%), non-categorical parameters: mean±standard deviation; ns = not significant;

1 hemagglutination inhibition (HI) antibody analysis (an SRH immunogenicity analysis was carried out in 58 (94%) subjects in each group since 2 samples per group were hemolytic);

2 two younger subjects were erroneously enrolled in the elderly age group but evaluated in the younger age group for the safety analysis.

### Safety and reactogenicity

All 126 subjects vaccinated were included in the safety evaluation. In the safety population, subjects were analyzed in 2 age groups according to their real age at the time of enrolment. No serious adverse events and no influenza-like illnesses (ILI) occurred during the study period.

**Figure 2:**
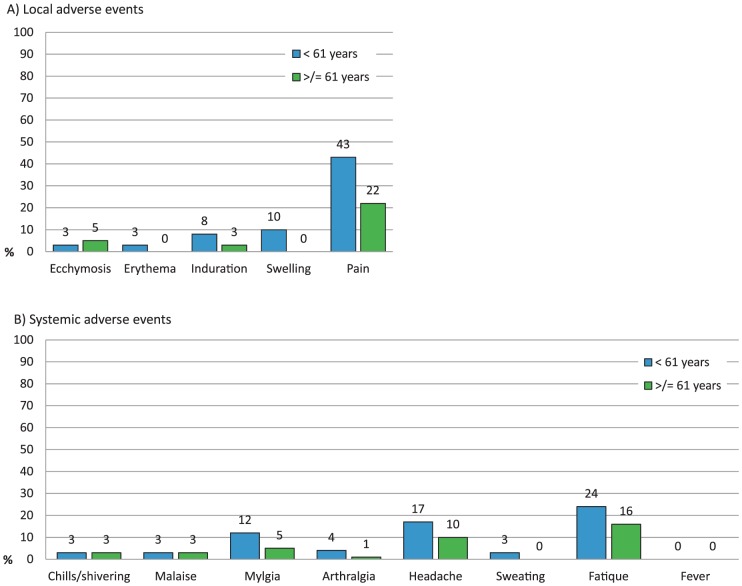
Percentage of younger (<61 years of age) and older (≥61 years of age) subjects who experienced solicited local (A) or systemic (B) adverse events. None had severe adverse reactions.

More non-elderly adults than elderly subjects reported solicited local or systemic reactions (62% vs. 44%, p<0.003). The most commonly reported reactions were pain at the injection site (33%) and fatigue (20%) following vaccination (figure 2). Pain was mild in 23 (37%) and moderate in 4 (6%) younger subjects as well as mild in 12 (20%) and moderate in 1 (2%) of the older subjects. The proportion of subjects with unsolicited AEs or local and systemic reactions continuing beyond 4 days after vaccination independent of relatedness to vaccination was not significantly different between the two groups at 8 (13%) elderly and 5 (8%) non-elderly adults. Three subjects had reactions at the injection site continuing after day 4, 2 had diarrhea and 2 had chills, all other AEs were each reported by one subject only (fatigue, malaise, tooth infection, injury, joint swelling, dizziness, headache and pain in the extremity).

### Immunogenicity

All but 6 vaccinated subjects were analyzed as part of the HI per protocol (PP) population and all but 10 as part of the SRH per protocol population. As the number of subjects in the PP population and the modified intention-to-treat (MITT) population differed by less than 10%, the immunogenicity analyses were carried out in the PP population as pre-specified in the analysis plan.

The proportion of the population with a hemagglutinin inhibition titer of 40 or more after vaccination for the three strains A(H1N1), A(H3N2) and B was 87%, 98% and 93% respectively, in adults below 61 years and 90%, 98% and 90% in adults of 61 years and older (table 3). The protection rate did not differ significantly between the elderly and the younger adults for any of the three strains. In the younger age group the CHMP criteria [6] were all met in the HI assay except with regard to the B strain, against which only 35% of subjects demonstrated seroconversion or a significant increase in antibody titers (table 4). In subjects aged 61 years and older, seroconversion or a significant increase in antibody titers was found in 27% for the A(H3N2) strain and 17% for the B strain.

**Table 3:** Vaccine immunogenicity assessed by HI assay - per protocol population.

	18–60 YOA (N = 60)							≥61 YOA (N = 60)						
Strains		A(H1N1)		A(H3N2)		B			A(H1N1)		A(H3N2)		B	
PRE-VACCINATION														
		n/N^1^	%	n/N	%	n/N	%		n/N	%	n/N	%	n/N	%
**GMT** 2		17		40		36			26		87		43	
**95% CI** 3		12–25		27–59		27–49			18–37		65–117		33–56	
**Seroprotection rate** 4		23/60	38%	35/60	58%	35/60	58%		26/60	43%	53/60	88%	42/60	70%
**95% CI** 3		26–52		45–71		45–71			31–57		77–95		57–81	

POST-VACCINATION														
	CHMP^7^	n/N	%	n/N	%	n/N	%	CHMP^7^	n/N	%	n/N	%	n/N	%
**Seroconv. rate** 5		21/29	72%	12/12	100%	5/7	71%		10/15	67%	1/1	100%	2/4	50%
**Significant increase** 6		20/31	65%	20/48	7342%	16/53	30%		31/45	69%	15/59	25%	8/56	14%
**Seroconv. rate or significant increase** 6	**>40%**	41/60	68%	32/60	53%	21/60	35%	**>30%**	4/60	68%	16/60	27%	10/60	17%
**95% CI**		55–80		40–66		23–48			55–80		16–40		8–29	
**GMT**		205		264		128			167		245		81	
**95% CI**		137–308		201–347		99–166			115–242		184–327		64–103	
**GM Increase** 8	**>2.5**	12		6.61		3.54		**>2.0**	6.46		2.81		1.89	
**95% CI**		7.8–18		4.24–10		2.52–4.97			4.65–8.98		2.05–3.87		1.58–2.26	
**Seroprotection rate**	**>70%**	52/60	87%	59/60	98%	56/60	93%	**>60%**	54/60	90%	59/60	98%	54*60	90%
**95% CI**		75–94		91–100		84–98			79–96		91–100		79–96	

1 n/N: responders (n) as proportion of the (sub-)population (N).

2 GMT: geometric mean titer;

3 95% CI: 95% confidence interval.

4 Seroprotection rate: proportion of subjects with a protective titer pre- or post-vaccination (titer ≥40).

5 Seroconversion rate: proportion of subjects with antibody increase from <10 pre-vaccination to ≥40 post-vaccination.

6 Significant increase: proportion of subjects with an antibody titer of ≥10 pre-vaccination and 4-fold antibody increase post-vaccination.

7 CHMP criteria.

8 GM increase = geometric mean increase.

**Table 4:** Licensing criteria for influenza vaccine immunogenicity laid down by the European Committee for Medicinal Products for Human Use.

	Age	Age
Assessment	18–60 years	61 years and above
Proportion of subjects achieving seroconversion or a significant increase in anti-HA antibody titer^1^	>40%	>30%
Mean geometric increase	>2.5	>2.0
Proportion of subjects achieving an HI titer ≥40	>70%	>60%

1 Either a pre-vaccination HI titer<1∶10 and a post-vaccination HI titer ≥1∶40 or a pre-vaccination HI titer ≥1∶10 and a minimum four-fold rise in post-vaccination HI antibody titer.

Pre-vaccination HI GMTs were similar in non-elderly adults and elderly subjects, with a tendency towards higher pre-vaccination GMTs in elderly subjects. The mean GMT increase and the post-vaccination GMTs for all three strains were higher in non-elderly than in elderly subjects. Interestingly, post-vaccination GMTs were highest in the A/Perth/16/2009 (H3N2)-like strain for both age groups, and GM increase was highest for A(H1N1) antibodies.

Pre-vaccination antibody titers were protective for the A(H1N1), A(H3N2) and B influenza strains in 23/60 (38%), 35/60 (58%) and 35/60 (58%) younger adults respectively and in 26/60 (43%), 53/60 (88%) and 42/60 (70%) elderly subjects (table 3).

In the study population, 61% of the younger subjects and 91% of the older age group had had at least one previous seasonal influenza vaccine, the majority of subjects having received their last seasonal influenza vaccine the previous year (2010). Additionally, 13% of younger and 8% of elderly subjects had received a pandemic A(H1N1) vaccine in 2009. Among those individuals who had not previously received seasonal or pandemic influenza vaccines against the currently recommended strains, 10 of 33 (30%) in the younger age group and 1 of 8 (12.5%) in the older age group had protective H1N1 antibody titers before vaccination (table 5). Ten of 32 (31%) individuals in the younger age group had protective antibodies against A(H3N1) and 13 of 32 (41%) had protective antibodies against the B strain. Conversely, 6 of 9 (67%) elderly subjects who had not previously been vaccinated had protective antibodies against both the A(H3N1) and the B strain.

**Table 5:** Evaluation of the antibody titers to pandemic A(H1N1) antigen prior to vaccination in the intention-to-treat analysis.

	Age	Age
	18–60 years	61 years and above
Subjects with previous A(H1N1) vaccine^1^	29/62 (47%)	55/64 (86%)
Protective A(H1N1) antibody titer^2^ among all subjects	24/62 (39%)	27/64 (42%)
Protective A(H1N1) antibody titer^2^ without previous vaccine^1^	10/33 (30%)	1/8 (13%)
Protective A(H1N1) antibody titer^2^ with previous vaccine^1^	14/29 (48%)	26/55 (47%)

1 Vaccination containing pandemic A(H1N1) antigen: either 2009 pandemic A(H1N1) vaccine or the 2010/2011 northern hemisphere seasonal influenza vaccine.

2 pre-vaccination HI titer ≥40.

Of those individuals who had previously received a vaccine containing antigens resembling those of the current vaccine (i.e. the seasonal influenza vaccine for 2010/11 or the 2009 pandemic influenza vaccine for the A(H1N1) strain [9], [12]), 14 of 29 (48%) in the younger age group and 26 of 55 (47%) in the older age group had protective antibodies prior to vaccination (table 5). Of the younger previously vaccinated individuals, 26 of 30 (87%) had protective antibodies against A(H3N2) and 24 of 30 (80%) had protective antibodies against B. In the older age group protective antibodies were detected prior to immunization against A(H3N2) in 51 of 55 subjects (93%) and against the B strain in 40 of 55 subjects (73%).

## Discussion

This study was conducted to evaluate the safety, clinical tolerability and immunogenicity in younger and older adults of a trivalent inactivated surface antigen influenza vaccine produced in mammalian cell culture. The primary endpoint was to assess antibody levels against the three strains of influenza recommended by the WHO for the 2011/2012 Northern hemisphere influenza season [8].

In contrast to other influenza vaccines, this trivalent vaccine was produced in a mammalian cell line (Madin-Darby canine kidney (MDCK) cells) enabling a demand focused manufacturing process. The selection of this cell line and the production process have been assessed for possible risks one concern being a possible tumorigenic potential of the MDCK cells. This was shown not to be the case [13], The manufacturing process sufficiently removes cells from vaccine, and, in contrast to influenza vaccine production in embryonated eggs, the risk of contamination is significantly reduced with the use of MDCK cells [4]. With the egg-free production of influenza vaccines the risk of allergic reactions to egg antigen is eliminated and safety of this cell-derived vaccine has previously been demonstrated [3], [14].

Vaccine efficacy is not determinable prior to seasonal influenza epidemics since testing efficacy implies evaluating subjects who have been exposed to the virus. A meta-analysis has shown that the efficacy of vaccination is reduced if the strains circulating during the influenza season do not match the WHO recommended strains [15]. For feasibility reasons, the criteria released by the European Committee for Medicinal Products for Human Use for the annual evaluation of licensed influenza vaccines make use of surrogate markers of vaccine efficacy [6]. These criteria also take into account that immunological response to influenza vaccines is age-dependent, and require HI or SRH antibody testing along with an evaluation of adverse reactions. The parameters taken into account in determining vaccine response are: a) proportion of subjects achieving seroconversion or a significant increase in anti-HA antibody titers, b) mean GMT antibody or GMA increase, and c) proportion of subjects achieving an HI titer ≥40. In this study the immunogenicity criteria required were met in both age groups. However, the response to the B strain was lower than to the A strains in both age groups, as previously reported for other seasonal influenza vaccines [14]. Only 35% of the younger and 17% of the elderly subjects achieved seroconversion or a 4-fold or greater increase in B antibody titers. However, 93% of the younger adults and 90% of the elderly adults in this study developed protective antibody titers (titer ≥40) against the influenza B strain, meaning that the vaccine met the CHMP criteria for all three influenza strains.

Interestingly, the majority of subjects who had an influenza vaccine consisting of the same influenza antigens as in the previous year still had protective antibodies against the A(H3N2) and the B strain, but only 48% of the younger and 47% of the older adults were still protected against the A(H1N1) stain. This may imply that either antibody levels to A(H1N1) decrease at a faster pace, or that individuals did not develop a high enough antibody titer in the first place. Taking into account that the subjects in a study carried out last year with another influenza vaccine [10] did develop sufficient antibody titers, the main reason for the low antibody levels observed here is probably that A(H1N1) antibody titers decline faster. The fact that protective antibody titers from influenza vaccines received the previous year can only be detected in less than half of subjects makes it wise to recommend annual vaccination even if antigen composition has not changed from the previous season. Also, this finding requires further observation and study.

The vaccine was generally well tolerated and the number of adverse events did not exceed rates found in other studies of seasonal influenza vaccines [10], [16]. Adverse events were more often described by subjects younger than 61 years than by older subjects. Pain at the injection site was reported in 43% of younger adults vs. 22% of older adults, but none of the individuals developed severe symptoms. Systemic adverse reactions mainly consisted of fatigue, myalgia and headache – all solicited – during the first few days after vaccination.

The study population comprised a high percentage of previously vaccinated subjects, especially in the older age group, reflecting current vaccination guidelines in Germany [17], [18]. Of those subjects who had not been vaccinated against the three strains recommended for the 2010/2011 Northern hemisphere season, 9 of 32 from the younger age group (28%) and 1 of 8 (12.5%) from the older age group had protective antibody titers against A(H1N1), a seroprevalence rate in line with figures from the US [19] and Northern Germany [20].

In conclusion, the 2011/2012 composition of this cell-derived seasonal influenza vaccine proved to be safe and generated sufficient antibody titers.

## Supporting Information

Checklist S1 **CONSORT Checklist.**
(DOCX)

Protocol S1 **Trial protocol.**
(PDF)
