# Supplementary material for: Clinical Trial to Evaluate the Safety and Immunogenicity of a Trivalent Surface Antigen Seasonal Influenza Vaccine Produced in Mammalian Cell Culture and Administered to Young and Elderly Adults with and without A(H1N1) Pre-Vaccination
Source: PLoS One. 2013 Aug 16;8(8):e70866. doi: 10.1371/journal.pone.0070866 (PMC3745456; doi:10.1371/journal.pone.0070866)
Supplement: Protocol S1 — Trial protocol. (PDF) [file pone.0070866.s002.pdf]

PROTOCOL SYNOPSIS V58_25S Version 1

EUDRACT No 2010-024613-31

A Phase III Open Label, Uncontrolled, Multi Center Study to Evaluate Safety and Immunogenicity of a Surface, Antigen, Inactivated, Influenza Vaccine Produced in Mammalian Cell Culture (Optaflu®), Formulation 2011/2012, when Administered to adult and elderly subjects.

Property of Novartis Vaccines and Diagnostics

Confidential

May not be used, divulged, published or otherwise disclosed without written consent of Novartis Vaccines and Diagnostics.

PROTOCOL SYNOPSIS V58_25S VERSION 1

**Name of Sponsor**	**Protocol number:**	**Eudract number:**	**Date of Protocol Synopsis:**
Novartis Vaccines and Diagnostics	V58_25S	2010-024613-31	11Dec10

Title of Study: A Phase III Open Label, Uncontrolled, Multi Center Study to Evaluate Safety and Immunogenicity of a Surface, Antigen, Inactivated, Influenza Vaccine Produced in Mammalian Cell Culture (Optaflu®), Formulation 2011/2012, when Administered to adult and elderly subjects.

Publication (reference): None

**Study Period:** Each subject will participate for approximately 3 weeks after immunization in the study.	**Clinical Phase:** III

Rationale: Influenza poses a significant threat to individual and public health, and influenza vaccination with a trivalent inactivated influenza vaccine is widely recommended to children, adults at risk and elderly. Due to antigenic changes of influenza viruses, the virus strains used in interpandemic influenza vaccines are adjusted every year according to WHO (World Health Organization) and CHMP (Committee for Medicinal Products for Human Use) recommendations. Following a change in the vaccine antigen composition recommendation from the previous season, immunogenicity and tolerability of the newly composed vaccines are subject of evaluation in an annual clinical trial in non-elderly adult and elderly subjects according to the guidelines set by EMEA (CPMP/BWP/214/96).

Study Agent/Intervention Description: A single 0.5 mL dose of the cell derived subunit trivalent non-adjuvated influenza study vaccine will be supplied in prefilled syringes and will be administered intramuscularly in the deltoid muscle of (preferably) the non dominant arm.

Objectives:

Safety Objectives: To evaluate the safety of a single intramuscular (IM) injection of Optaflu in adult and elderly subjects in compliance with the requirements of the current EU recommendations for clinical trials related to yearly licensing of influenza vaccines (CPMP/BWP/214/96).

Immunogenicity Objectives:

Primary

To evaluate the antibody response to each influenza vaccine antigen, as measured by

**Name of Sponsor** Novartis Vaccines and Diagnostics	**Protocol number:** V58_25S	**Eudract number:** 2010-024613-31	**Date of Protocol Synopsis:** 11Dec10
hemagglutination inhibition (HI) at 21 days post-immunization in adult and elderly subjects in compliance with the requirements of the current EU recommendations for clinical trials related to yearly licensing of influenza vaccines.<br><br>Antibodies maybe additionally quantified using the Single Radial Hemolysis (SRH) test for confirmation purposes. (Note for Guidance on Harmonization of Requirements for Influenza Vaccines. CPMP/BWP/214/96: 12 March 1997).			
**Methodology:** In this open label single treatment arm study, 126 subjects will be enrolled into two groups according to age (at least 50 subjects aged 18-60 years should be evaluable; at least 50 subjects aged over 60 years should be evaluable). On Day 1, the study staff will query each female of childbearing potential to determine the date of her last menstrual period and, the subject's commitment to use a birth control from Day 1 up to and including the three weeks following vaccination. To be eligible for this study, all females of childbearing potential will be required to have a negative urine pregnancy test to receive study vaccination. Subjects will be observed for approximately 30 minutes after study vaccination on Day 1 for any immediate reactions. Each subject will be instructed to complete a diary card for 3 days post the day of immunization to describe local (pain, erythema, ecchymosis, swelling and induration) and systemic reactions [fever (i.e., axillary temperature ≥ 38°C), chills/shivering, malaise, headache, myalgia, arthralgia, sweating and fatigue]. Subjects will be contacted by phone on Day 5 (window: 0/+3) after immunization to ensure that local and systemic reaction data have been collected on the subject's Diary Card and also to determine the subject's clinical status. All adverse events will be collected during Days 1 to 4. All adverse events necessitating a physician's visit or consultation and/or leading to premature study discontinuation and all serious adverse events will be collected throughout the trial. Subjects will be informed that in the event of severe inter-current infection (i.e., any severe flu like symptoms) during the study period until Day 22 (window: -1/+5), he/she must contact the Investigator who will take a nasal and/or pharyngeal swab for the diagnosis of influenza or any other respiratory infection of viral origin. Specimens will be analyzed via Quick test and RT-PCR or culture for confirmatory purposes.<br><br>Blood samples for immunogenicity assays will be collected before vaccination (Day 1) and 21 days after vaccination (Day 22, window: -1/+5).			
**Number of Subjects planned:** Approximately 126 subjects are to be enrolled, of which 63 in the non-elderly adult age group (age 18 to 60 years) and 63 in the elderly age group (age over 60 years). This sample size allows for up to 13 non evaluable subjects per group (Non-evaluable subjects are enclosed in the Per Protocol Set exclusions due to protocol deviation as predefined in the analysis plan). In the non-elderly adult age			

**Name of Sponsor** Novartis Vaccines and Diagnostics	**Protocol number:** V58_25S	**Eudract number:** 2010-024613-31	**Date of Protocol Synopsis:** 11Dec10
group, no more than approximately half of the subjects should be between 41 and 60 years of age.<br>Subjects who received the immunization and provided post-baseline safety data will be included in the safety analyses. Subjects with major protocol deviations will not be included in the per protocol immunogenicity analyses.			
**Subject Population:** The study population will consist of healthy, adult volunteers 18 years of age or older, male and female who have not received any seasonal or pandemic influenza vaccine or have not had a laboratory confirmed seasonal or pandemic influenza disease within the past 6 months.			
**Subject Characteristics and Main Criteria for Inclusion and Exclusion:**<br><br>Inclusion Criteria: Healthy, adult volunteers (age 18 to 60 years) and elderly volunteers (age over 60 years).<br><br>Exclusion Criteria: Individuals with any serious chronic or acute disease or known or suspected impairment of the immune system.<br><br>The full list of inclusion and exclusion criteria based on this summary is included in protocol section 4.0.			
**Vaccines:** <u>Test Vaccine:</u> Optaflu<br>A single 0.5 mL dose of the study vaccine will be supplied in prefilled syringes and will be administered intramuscularly in the deltoid muscle of (preferably) the non dominant arm.<br><br>A 0.5 mL dose of Optaflu contains purified viral envelope-glycoproteins neuraminidase (NA) and hemagglutinin (HA), including 15 µg of HA of each of the three strains (A/H1N1-like strain, A/H3N2-like strain, B-like strain), recommended for inclusion in the vaccine composition for the influenza season 2011/2012 in the Northern Hemisphere.			

**Name of Sponsor** Novartis Vaccines and Diagnostics	**Protocol number:** V58_25S	**Eudract number:** 2010-024613-31	**Date of Protocol Synopsis:** 11Dec10

Immunogenicity Endpoints

The following serological assessments will be considered for each strain in non-elderly adult subjects, aged between 18 and 60, and at least one of the assessments should meet the indicated requirements:

- The proportion of subjects achieving seroconversion or significant increase in HI titer or SRH area > 40%
- Mean geometric increase > 2.5
- The proportion of subjects achieving an HI titer ≥ 40 or SRH area ≥ 25 mm^2 should be > 70%

The following serological assessments will be considered for each strain in elderly subjects, aged 61 years and over, and at least one of the assessments should meet the indicated requirements:

- Proportion of seroconversion or significant increase in HI titer or SRH area > 30%
- Mean geometric increase > 2.0
- The proportion of subjects achieving an HI titer ≥ 40 or SRH area ≥ 25 mm^2 should be > 60%

Circulating anti-HA antibodies will be measured by HI and possibly SRH assay just prior to vaccination (Day 1) and approximately 3 weeks after the vaccination (Day 22). For the purposes of calculation, any HI result < 10 (i.e. undetectable) will be expressed as 5, and any negative SRH result will be expressed as 4 mm^2.

In HI tests, seroconversion or significant increase in antibody titer corresponds to:

- negative pre-vaccination serum / post-vaccination serum titer ≥ 40 or
- at least a four-fold increase in titer from positive pre-vaccination serum

In SRH tests, seroconversion or significant increase in antibody titer corresponds to:

- negative pre-vaccination serum / post-vaccination serum area ≥ 25 mm^2
- at least a 50% increase in area from positive pre-vaccination serum

**Name of Sponsor** Novartis Vaccines and Diagnostics	**Protocol number:** V58_25S	**Eudract number:** 2010-024613-31	**Date of Protocol Synopsis:** 11Dec10
**Safety Endpoints**<br><br>Safety will be assessed in accordance with available safety data on influenza vaccines:<br><br>• Local and systemic reactions will be assessed for 3 days post the day of vaccination<br><br>AEs Days 1 to 4. All AEs necessitating a physician's visit or consultation and/or leading to premature study discontinuation and all SAEs until Day 22 of until resolution.			
**Interim Analysis:** No interim analysis of data from this trial is planned. Should it later become necessary, the analysis will be governed by the procedures specified in the Novartis Vaccines SOP entitled "Interim Analysis".			
**Data Monitoring Committee:** A data monitoring committee will not be utilized for this study.			

TABLE OF CONTENTS

PROTOCOL SYNOPSIS V58_25S VERSION 1 2
TABLE OF CONTENTS 7
LIST OF ABBREVIATIONS AND DEFINITIONS OF TERMS 10
1.0 BACKGROUND AND RATIONALE 11
2.0 OBJECTIVES 13
3.0 STUDY DESIGN AND INVESTIGATIONAL PLAN 13
3.1 Overview of Study Design 13
3.2 Discussion of Overall Study Design 14
3.3 Study Procedures and Flowchart 14
3.3.1 Subject Numbering 21
3.3.2 Blinding procedures 21
3.3.3 Vaccine Supply, Storage, Tracking and Labeling 21
3.3.4 Processing, Labeling and Storage of Serum Samples for Serology 22
3.4 Duration of Subject's Expected Participation in the Entire Study 23
3.5 Stopping/Pausing Rules 23
4.0 SELECTION OF STUDY POPULATION 24
4.1 Inclusion Criteria 24
4.2 Exclusion Criteria 24
4.3 Withdrawal of Subjects from Therapy or Assessment 26
5.0 TREATMENT OF SUBJECTS 27
5.1 Investigational Vaccine 27
5.2 Control Vaccines 28
5.3 Concomitant Vaccines or Treatment 28
5.4 Vaccines Preparation and Administration 28
5.5 Other Concomitant Treatment or Vaccines 29
5.6 Vaccination Compliance 30
6.0 EFFICACY/IMMUNOGENICITY AND SAFETY ASSESSMENTS 30
6.1 Appropriateness of Measurements 30

6.1.1 Immunogenicity 30
6.1.2 Methods, Criteria and Timing for Assessing and Recording Efficacy/Immunogenicity Parameters 30
6.2 Safety Parameters 31
6.2.1 Local and Systemic Reactions 31
6.2.2 Adverse Events 31
6.2.3 Serious Adverse Events 32
6.2.4 Pregnancies 33
6.2.5 Methods and Timing for Assessing and Recording Safety Parameters 33
6.3 Data Monitoring Committee 35
7.0 STATISTICAL PLAN 35
7.1 Statistical Hypothesis 35
7.2 Sample Size and Power Considerations 35
7.3 Population for Analysis 35
7.4 Analysis of Demographic and Baseline Characteristics 36
7.5 Analysis of Efficacy/Immunogenicity Endpoints 36
7.5.1 Description of Response Variables 36
7.5.2 Statistical Methods for Efficacy/Immunogenicity Variables 37
7.6 Analysis of Safety (Endpoints) and Tolerability 38
7.6.1 Analysis of Extent of Exposure 38
7.6.2 Analysis of Local and Systemic Reactions 38
7.6.3 Analysis of Other Adverse Events 39
7.7 Planned Interim Analysis 39
8.0 STUDY MONITORING, AUDITING AND DOCUMENTATION 39
8.1 Study Monitoring 39
8.2 Source Data Verification 40
9.0 DATA MANAGEMENT 40
9.1 Data Handling Procedures 41
9.2 Documentation of Study Findings 41
9.3 Data Protection 41
10.0 RECORD RETENTION 42

11.0 USE OF INFORMATION AND PUBLICATION 42
12.0 ETHICS 43
12.1 Regulatory and Ethical Compliance 43
12.2 Informed Consent Procedures 43
12.3 Responsibilities of the Investigator and IRB/IEC/REB 43
12.4 Protocol Adherence 44
12.5 Protocol Amendments 44
13.0 REFERENCE LIST 45

LIST OF ABBREVIATIONS AND DEFINITIONS OF TERMS

Abbreviations

ACIP	Advisory Committee on Immunization Practices
AE	Adverse Event
AP	(Statistical) Analysis Plan
BCDM	Biostatistics and Clinical Data Management
B&SR	Biostatistics and Statistical Reporting
CRF	Case Report Form
DCF	Data Clarification Form
EC	Ethics Committee
FAS	Full Analysis Set
GCP	Good Clinical Practice
CHMP	Committee for Medicinal Products for Human Use
GMC	Geometric Mean Concentration
GMP	Good Manufacturing Practice
GMR	Geometric Mean Ratio
GMT	Geometric Mean Titer
HEE	Hidden Entry Envelopes
HI	Haemagglutination Inhibition
HIPAA	Health Insurance Portability and Accountability Act
ICD-9	International Classification of Diseases Ninth Edition
ICF	Informed Consent Form
ICH	International Conference on Harmonization
IM	Intramuscular
IRB	Institutional Review Board
ITT	Intention-To-Treat
MITT	Modified Intention-To-Treat
LSLV	Last Subject Last Visit
MedDRA	Medical Dictionary for Regulatory Activities
PP	Per Protocol
REB	Regional Ethics Board
SAE	Serious Adverse Event
SBA	Serum Bactericidal Assay
SOC	System Organ Class
SOP	Standard Operating Procedure
WHO	World Health Organization

1.0 BACKGROUND AND RATIONALE

Surface, Antigen, Inactivated, Influenza Vaccine Produced in Mammalian Cell Culture (Optaflu®) currently marketed in Europe by Novartis Vaccines is propagated in mammalian cell cultures, inactivated and purified.

Influenza is still a major health concern in the world and is one of the leading causes of death [1]. About half a million people have died during influenza outbreaks in the past 20 years. Studies of morbidity and mortality associated with influenza indicate that hospitalization rates for adults with high-risk medical conditions often increase five-fold during epidemics, leading to an average of 172,000 excess hospitalizations during each epidemic. [2]. This has important economic consequences with high annual productivity loss in the industrialized nations [3]. In addition, influenza virus continues to be an important cause of respiratory infection and an important contributor to morbidity in at-risk populations [4]. Typical influenza illness is an acute febrile disease attended by respiratory symptoms, myalgia, and headache. Unlike many other common respiratory infections, it can cause extreme malaise lasting several days. A non-productive cough, stemming from destruction of tracheal epithelium, is almost invariable. More severe illness can result if influenza virus invades the lungs (primary viral pneumonia) or if secondary bacterial pneumonia occurs.

The highest attack rates for influenza occur in children (40 percent in preschool children and 30 percent in school-age children) [5-7], who are also major disseminators of influenza viruses in the community, because they are the main channel through which influenza is introduced into households [8-10]. As a consequence, outbreaks in children are usually soon followed by the occurrence of influenza-like illnesses among adults and elderly people. Elderly persons and those with underlying chronic disease are at increased risk for complications of influenza infection [11]. The significant increase seen in mortality during an influenza epidemic is a direct result not only of pneumonia but also of cardiopulmonary or other chronic diseases that can be exacerbated by influenza infection.

The virus strains responsible for influenza vary in an unpredictable fashion, so that the type of virus as well as the magnitude of influenza activity change from one winter to the other [12]. This change confers a selective advantage to the new virus subtypes because large segments of the population have not developed effective antibodies and are thus susceptible to the infection. The high genetic mutability of the influenza virus requires a continuous monitoring of the influenza epidemic by national surveillance systems so to identify the prevalent virus antigens in the population [13]. Following the recommendations of these surveillance systems, every year anti-influenza vaccines are produced ad hoc and distributed worldwide to control the new variants of the influenza virus.

Although new antiviral agents have improved the ability to treat influenza infections, vaccination remains the most important method of controlling influenza through

prevention. Influenza vaccines offer cost-effective protection against the disease and its complications [14-20], and for this reason they are recommended and widely used every year prior to the expected flu outbreaks [21].

Since their development inactivated influenza vaccines have been produced in the allantoic cavity of embryonated hen eggs. However, the efficiency of the method is limited. In contrast, the new production methods on mammalian cell lines reduce reliance on the supply of embryonated eggs and generate more flexibility, adequate availability of substrate for virus growth and the possibility of significant higher virus yields [24]. In addition the cell-derived vaccines do not require extensive advance planning and can be produced rapidly, to a large extent in the event of an emerging pandemic [25].

Novartis Vaccines has therefore adopted influenza vaccine produced in a specifically developed cell line cloned from Madin Darby Canine Kidney (MDCK) tissue; the influenza viruses are expanded in these high concentration cell cultures and then harvested to produce the investigational vaccine. Previous conducted clinical trials show that the subunit influenza vaccine produced in mammalian cell culture (Optaflu®) is generally safe and well tolerated in the study subjects and is immunogenic for all vaccine strains.

The previous clinical data of Optaflu clearly indicates a good safety/tolerability profile. The immunogenicity was evaluated by Haemagglutination Inhibition (HI) test and the results met the criteria established in March 1992 by the Ad Hoc Working Party on Biotechnology/Pharmacy [22] and formalized in March 1997 by the CPMP [23] with respect to the number of seroconversions, mean geometric increase and proportion of subjects with a titer of antibodies after vaccination, thereby showing the vaccine to be effective in prevention of influenza.

This protocol is designed to evaluate the safety, clinical tolerability and immunogenicity of Optaflu, Northern Hemisphere formulation 2011/2012.

The principal aim is to provide safety/immunogenicity data, in compliance to current EU Guidelines [23], with the intent of obtaining marketing approval of the vaccine formulation intended for use prior to the next influenza season.

A comprehensive review of Optaflu is contained in the Investigator's Brochure or Summary of Product Characteristics supplied by Novartis Vaccines; this document should be reviewed prior to initiating the study.

The trial will be conducted in compliance with the protocol, GCP and the applicable regulatory requirements.

2.0 OBJECTIVES

Objectives:

Safety Objectives:

To evaluate the safety of a single intramuscular (IM) injection of Optaflu in adult and elderly subjects in compliance with the requirements of the current EU recommendations for clinical trials related to yearly licensing of influenza vaccines (CPMP/BWP/214/96).

Immunogenicity Objectives:

<u>Primary</u>

To evaluate the antibody response to each influenza vaccine antigen, as measured by hemagglutination inhibition (HI) at 21 days post-immunization in adult and elderly subjects in compliance with the requirements of the current EU recommendations for clinical trials related to yearly licensing of influenza vaccines.

Antibodies maybe additionally quantified using the Single Radial Hemolysis (SRH) test for confirmation purposes. (Note for Guidance on Harmonization of Requirements for Influenza Vaccines. CPMP/BWP/214/96: 12 March 1997).

3.0 STUDY DESIGN AND INVESTIGATIONAL PLAN

3.1 Overview of Study Design

This is a single treatment arm, open label study complying with current CHMP requirements (CPMP/BWP214/96) for yearly evaluation of licensed flu vaccines.

Approximately 126 subjects are to be enrolled, of which 63 in the non-elderly adult age group (age 18 to 60 years) and 63 in the elderly age group (age over 60 years). This sample size allows for up to 13 non evaluable subjects per group (Non-evaluable subjects are enclosed in the Per Protocol Set exclusions due to protocol deviation as predefined in the analysis plan). In the non-elderly adult age group, no more than approximately half of the subjects should be between 41 and 60 years of age. On Day 1, the study staff will query each female of childbearing potential to determine the date of her last menstrual period and, the subject's commitment to use a birth control for the from Day 1 up to and including the three weeks following vaccination. To be eligible for this study, all females of childbearing potential will be required to have a negative urine pregnancy test to receive study vaccination. Subjects will be observed for approximately 30 minutes after study vaccination on Day 1 for any immediate reactions. Each subject will be instructed to complete a diary card for 3 days post the day of vaccination to describe local (pain, erythema, ecchymosis, swelling and induration) and systemic reactions [fever (i.e., axillary temperature ≥ 38°C), chills/shivering, malaise, headache, myalgia, arthralgia,

sweating, fatigue]. Subjects will be contacted by phone on Day 5 (window: 0 / +3 days) after immunization to ensure that local and systemic reaction data have been collected on the Subject's Diary Card and also to determine the subject's clinical status. All adverse events will be collected during Days 1 to 4. All adverse events necessitating a physician's visit or consultation and/or leading to premature study discontinuation and all serious adverse events will be collected throughout the trial.

Subjects will be informed that in the event of moderate to severe inter-current infection (i.e., any moderate to severe flu like symptoms) during the study period until Day 22 (window: -1/+5), he/she must contact the Investigator who will take a nasal and/or pharyngeal swab for the diagnosis of influenza or any other respiratory infection of viral origin. Specimens will be analyzed via Quick test and RT-PCR or culture for confirmatory purposes. This will be recorded on the "Comments" Case Report Form; the swab and blood samples will be sent for analysis to appropriately qualified laboratories.

Blood samples for immunogenicity assays will be collected before vaccination (Day 1) and after 21 (Day 22, window: -1/+5) days.

3.2 Discussion of Overall Study Design

This protocol is designed to evaluate the safety, clinical tolerability and immunogenicity Optaflu, Northern Hemisphere formulation 2011/2012.

The principal aim is to provide safety/immunogenicity data, in compliance to current EU Guidelines [23], with the intent of obtaining marketing approval of the vaccine formulation intended for use prior to the next influenza season.

3.3 Study Procedures and Flowchart

Informed consent must be obtained from the subject, or where applicable, the subject's legally acceptable representative(s) prior to the performance of any trial specific tests or evaluations, i.e., any unusual or non-routine procedures that involve risk, however trivial, to the subject.

Visit 1, Day 1: Vaccination:

- Explain and obtain written informed consent from the subject. Informed consent must be obtained prior to performance of any study-specific tests or evaluations.
- Obtain and record medical history and assess concomitant medication. On Day 1, the study staff will query each female of childbearing potential to determine the date of her last menstrual period and, the subject's commitment to use a reliable birth control from Day 1 up to and including the three weeks following vaccination. Adequate contraception is defined as hormonal (e.g., oral, injection, transdermal patch, implant, cervical ring), barrier (e.g., condom with spermicide

or diaphragm with spermicide), intrauterine device (IUD), or monogamous relationship with vasectomized partner who has been vasectomized for 6 months or more prior to the subject's study entry;

- For females of childbearing potential, perform a urine pregnancy test and document the result on the subject's source data sheet or medical record.

- If subject continues to meet all inclusion and no exclusion criteria, perform brief physical examination, and assign subject number for study.

- Record abnormalities from physical examination on the "Medical History" eCRF.

- Collect pre-vaccination blood sample (10 mL). See Section 6.1.1. For further information on blood collection.

- Measure body (preferably axillary) temperature immediately prior to vaccination.

- Administer the injection of Optaflu in the deltoid muscle of the (preferably) non dominant arm. [See section 5.4].

- Subjects will be observed in the clinic for approximately 30 minutes after study vaccination for any immediate reactions.

- Instruct the subject in the evaluation of local and systemic reactions, and recording of temperature.

- Dispense the subject's diary card for immunization reactions, and give instructions for its completion starting on the day of vaccination (after 6 hours) and for each of the following 3 days (i.e, Day 2 to Day 4).

- Subjects will be informed that in the event of severe inter-current infection (i.e., any severe flu like symptoms) during the study period until 21 days after vaccination (window: -1/+5), he/she must contact the Investigator who will take a nasal and/or pharyngeal swab for the diagnosis of influenza or any other respiratory infection of viral origin. Specimens will be analyzed via Quick test and RT-PCR or culture for confirmatory purposes. Inter-current infection will be recorded as an AE and documented in the eCRF.

- Schedule next clinic visit.

Visit 2, Day 5 (window: 0 / +3 days): Follow-up Telephone Contact:

- A telephone call will be made on Day 5 (window: 0 / +3 days) to assess local and systemic reactions, occurrence of adverse events, use of analgesics/antipyretics and prescription medications and to ensure that those data have been collected on the Subject's Diary Card.

Visit 3, Day 22 (window: -1/+5 days): Blood Collection and Final Clinic Visit (Study Termination - 3 weeks after the vaccination ;)

- Obtain interim history (with reconciliation of subject's diary card data) including local and systemic reactions, body (preferably axillary) temperature, adverse events and prescription medications.

- Perform brief physical examination, with check of the injection site.

- Take blood sample for immunogenicity assays (10 mL). See Section 6.1.1 for further information on blood collection.

- Complete study termination information.

Diagram 3.1.1 – Study Flow Diagram

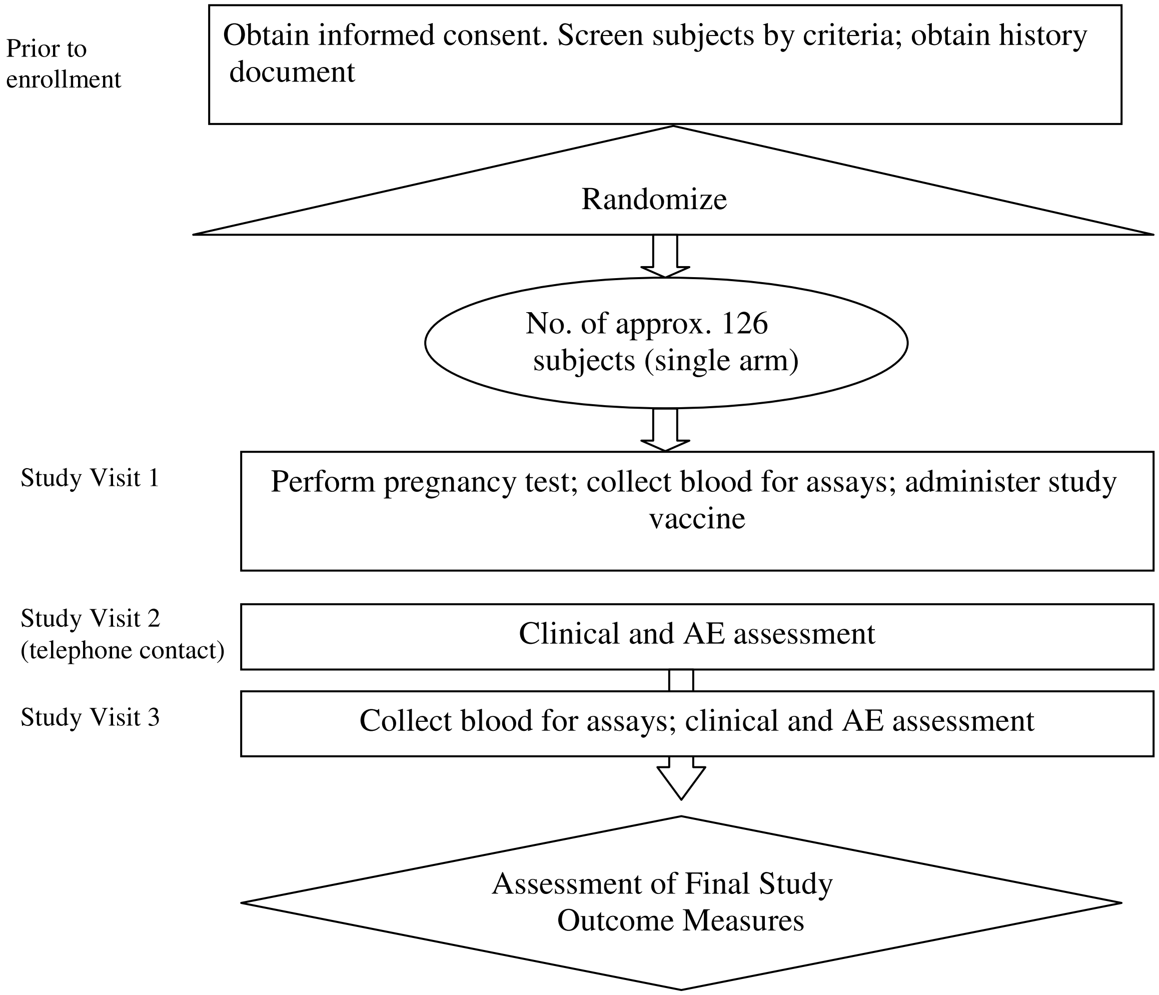

Table 3.1 - Times and Events Table

**Study Periods**	**Visit 1**	**Visit 2**	**Visit 3**
**Clinic Visit? (Yes/No)**[a]	Yes	No	Yes
**Study Day**	1	5	22
**Study Visit Window**	n/a	0/+3	-1/+5
ICF	x		
Exclusion/Inclusion	x[b]		
Medical history	x[b]		
Physical exam/ assessment[c]	x		x
Pregnancy Test (urine beta-human chorionic gonadotropin (ß-hCG) test)	x[b]		
**Investigational vaccine administered**	x		
Serology Blood draw [10 mL]	x[b]		x
Diary Card Dispensed [d]	x		
Diary Card Collected and/or Reviewed [d]		x	x
Assess Local/Systemic Reactions[e]	x	x	
Assess AEs and SAEs [f]	x	x	x
Concomitant medications	x	x	x
Study Termination			x

a. Clinic visit “no” refers to telephone contact only with subject
b. Performed prior to vaccination
c. Physical examination must be performed by a qualified health professional designated within the Site Responsibility Delegation Log. Brief physical exam will be performed at study Day 1 and 22. Physical examination of injection site and complaint-focused physical examination will be performed at visits on Day 1 and 22
d. Diary card review will be performed over the phone for Day 5 visit. Diaries will be returned at Day 22 visit.
e. Data on local and systemic reactions will be observed by the study personnel for all subjects for approximately 30 minutes after study vaccination. Subjects will record local and systemic reactions on the diary card daily for 3 days after the day of study vaccination

f. All the adverse event will be collected during Days1 to 4. All adverse events necessitating a physician's visit or consultation and /or leading to premature study discontinuation and all serious adverse event will be collected throughout the trial.

Diagram 3.1.2 - Study Procedure Flowchart - detailed

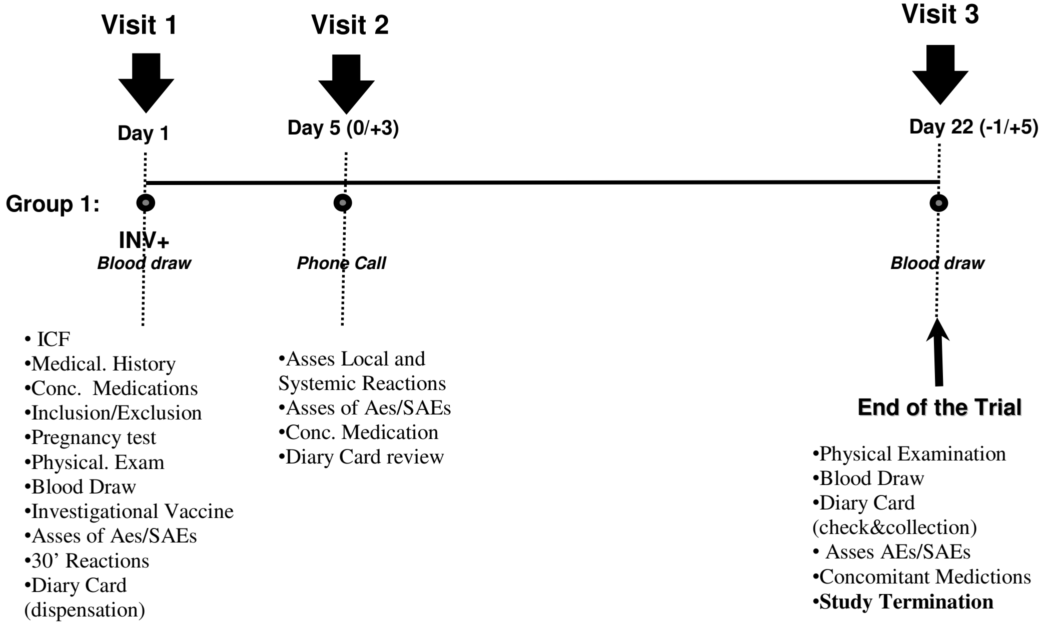

3.3.1 Subject Numbering

In agreement with GCP, each subject will be unambiguously identified by a code, which allows the identification of all the data reported for each subject. In Novartis Vaccines and Diagnostics, the code is a 5-digit number resulting from the combination of the site number and the subject number. The site number is assigned by Novartis Vaccines and Diagnostics to the investigative site. The first digit identifies the study site and the second digit the satellite (i.e. 12 is assigned to satellite 2 of the site 1). The next three digits identify the subject within the site and will be assigned sequentially, with 001 corresponding to the first subject enrolled in the 18-60 years age group and with 101 corresponding to the over 60 years age group at any particular site.

The subject number is assigned by the investigator upon signing the informed consent form and confirming the eligibility criteria.

Once assigned to a subject a number cannot be reused.

The investigator must keep track of the names of the subjects enrolled and their identifying number in a Subject Identification Code List.

3.3.2 Blinding procedures

The trial is designed as a single-arm, open-label study; both the study personnel and the subject will know which vaccine is being administered.

3.3.3 Vaccine Supply, Storage, Tracking and Labeling

The sponsor will supply the investigational test vaccine. Concomitant vaccines will not be provided by the Sponsor.

The investigator should acknowledge receipt of the investigational study vaccines. Study vaccines must be received by a designated person at the study site, handled and stored safely and properly, and kept in a secured location to which only the investigator or designee have access. Upon receipt, investigator or designee should ensure study vaccines are received in good condition. The vaccines at the site must not be used before the appropriate shipping conditions have been checked and confirmed by sponsor to be authorized for use. Study vaccine will be labeled and will comply with the legal requirements of each Country. All study vaccines should be stored according to the instructions specified on the labels.

Vaccines that have been stored differently from the manufacturer's indications **must not** be used unless the sponsor provides written authorization for use. In the event that the use cannot be authorized, vaccine supply must be replaced with fresh stock supplied by the sponsor. Batches of investigational vaccines must be stored separately from normal

hospital/practice stocks to prevent unintentional use of investigational products outside of the clinical trial setting.

The investigator should ensure that the investigational product(s) delivered to the site is used only in accordance with the approved protocol. Monitoring of drug accountability will be performed by the study monitor during site visits and at the completion of the trial.

The investigator should maintain an accurate record of products delivery to the site, the inventory at the site, the administration to the subjects, and the return to the sponsor of unused study vaccines. These records should include dates, quantities, batch number, expiration dates and the unique identifying number assigned to the investigational product and the subject.

A detachable label will be found either on the outer box or on the label of the primary container of the investigational product. The detachable label contains a unique identifier for each dose. The investigator must stick the detachable part on the administration log upon dispensing of the vaccine to certify that the vaccine was effectively administered and for tracking purposes.

At the conclusion of the study, and as appropriate during the course of the study, the investigator should return all unused study vaccines, packaging and supplementary labels to Novartis Vaccines and Diagnostics.

If the unused study vaccines are disposed at the site, the investigator should provide a copy of the site's procedure for destruction of hazardous material and documentation of the destruction. The following information must be included in the documentation of destruction:

Lot number

- Number of doses
- Date of destruction
- Destruction code (if available)
- Method of destruction
- Destruction performed by

3.3.4 Processing, Labeling and Storage of Serum Samples for Serology

10mL of blood sample should be drawn in order to obtain the minimum required serum volume for Immunological assays (HI and/or SRH). Blood will be drawn at Visit 1 prior to vaccination and at Visit 3 (day 22 -1/+5) for all subjects.

The blood will be centrifuged on the same day and the serum will be distributed in 2 aliquots using the tubes provided giving an "original" and a "duplicate" sample. "Original" and "duplicate" aliquots will be stored in separate storage boxes. The aliquots will be stored at a temperature of minus 18°C or below. At least 2 mL serum should be available for assays.

Each serum tube will be labeled with an identifying bar code including subject number, protocol number and visit number. Serum samples will be sent to the sponsor or will be collected by a representative of the sponsor.

For the labeling a set of serum tube and shipping log labels will be provided to the Investigator containing a uniquely identifying barcode with the following information preprinted: 1) protocol number for which a maximum of 10 digits are assigned; 2) the visit number followed by "S" indicating a serum sample; 3) the subject number that includes the coding for the site. The subject code requires to be added to the label by handwriting. The "original" or "duplicate" status of a sample is not encoded on the bar code and is printed in addition on the label. The serum tube labels are placed lengthwise on the tube prior to the serum separation.

For each shipment the shipping log an inventory of the samples which accompanies the shipment must be prepared. The information requested on the shipping log must be completed by a responsible person at the site shortly before the shipment is initiated. A copy of the shipping log has to be placed into the investigators study file.

Detailed instructions for labeling, storage and shipping of serum samples are included in the Serology Manual, provided separately.

Samples will be retained in accordance with regulatory guidance for retention of essential study documents described in section 10, provided that the integrity of the sample permits.

3.4 Duration of Subject's Expected Participation in the Entire Study

Each subject will participate approximately for 3 weeks after immunization into the study.

3.5 Stopping/Pausing Rules

There are no predetermined stopping rules other than those described in section 4.3.

The sponsor, or the investigator (following consultation with the sponsor) has the right to discontinue this study at any time. If the clinical study is prematurely terminated, the investigator is to promptly inform the study subjects and should assure appropriate therapy and follow-up for the subjects. All procedures and requirements pertaining to the

archiving of the documents should be followed. All other study materials (study medication/vaccines etc.) must be returned to the sponsor.

4.0 SELECTION OF STUDY POPULATION

4.1 Inclusion Criteria

1. Males and females volunteers of 18 years of age or older, mentally competent, willing and able to give written informed consent prior to study entry;
2. Individuals able to comply with all the study requirements;
3. Individuals in good health as determined by the outcome of medical history, physical examination and clinical judgment of the investigator.

Written informed consent must be obtained for all the subjects before enrollment into the study after the nature of the study has been explained.

4.2 Exclusion Criteria

1. Individuals with behavioral or cognitive impairment or psychiatric disease that, in the opinion of the investigator, may interfere with the subject's ability to participate in the study.
2. Individuals with any serious chronic or acute disease (in the judgment of the investigator), including but not limited to:<br>Medically significant Cancer (except for benign or localized skin cancer, cancer in remission for ≥10 years or localized prostate cancer that has been clinically stable for more than 2 years without treatment)<br>Medically significant advanced congestive heart failure (ie. NYHA class III and IV)<br>Chronic obstructive pulmonary disease (COPD);<br>Autoimmune disease (including rheumatoid arthritis, except for Hashimoto's thyroiditis that has been clinically stable for ≥ 5 years)<br>Diabetes mellitus type I;<br>Poorly controlled diabetes mellitus type II;<br>Advanced arteriosclerotic disease;<br>History of underlying medical condition such as major congenital abnormalities requiring surgery, chronic treatment, or associated with developmental delay (e.g.,

	Down's syndrome);<br>Acute or progressive hepatic disease;<br>Acute or progressive renal disease;<br>Severe neurological (es. Guillain–Barré syndrome) or psychiatric disorder;<br>Severe asthma
3.	Individuals with history of any anaphylactic reaction and/or serious allergic reaction following a vaccination, a proven hypersensitivity to any component of the study vaccine (e.g. influenza viral protein, and exipients);
4.	Individuals with known or suspected (or have a high risk of developing) impairment/alteration of immune function (excluding that normally associated with advanced age) resulting, for example, from:<br>receipt of immunosuppressive therapy (any parenteral or oral corticosteroid or cancer chemotherapy/radiotherapy) within the past 60 days and for the full length of the study;<br>receipt of immunostimulants;<br>receipt of parenteral immunoglobulin preparation, blood products and/or plasma derivates within the past 3 months and for the full length of the study;<br>suspected or known HIV infection or HIV-related disease;
5.	Individuals with known or suspected history of drug or alcohol abuse
6.	Individuals with a bleeding diathesis or conditions associated with prolonged bleeding time that in the investigator's opinion would interfere with the safety of the subject
7.	Female who are pregnant or nursing (breastfeeding) mothers or females of childbearing potential do not plan to use acceptable birth control measures, for the whole duration of the study. Adequate contraception is defined as hormonal (e.g., oral, injection, transdermal patch, implant, cervical ring), barrier (e.g., condom with spermicide or diaphragm with spermicide), intrauterine device (IUD), or monogamous relationship with vasectomized partner who has been vasectomized for 6 months or more prior to the subject's study entry
8.	Individuals who are not able to comprehend and to follow all required study procedures for the whole period of the study
9.	Individuals with history or any illness that, in the opinion of the investigator, might interfere with the results of the study or pose additional risk to the subjects due to participation in the study.

10. Individuals Within the past 6 months, they have:<br>had any seasonal or pandemic laboratory confirmed influenza disease;<br>received any seasonal or pandemic influenza vaccine;
11. Individuals with any acute or chronic infections requiring systemic antibiotic treatment or antiviral therapy within the last 7 days
12. Individuals that have experienced fever (i.e., axillary temperature ≥ 38°C) within the last 3 days of intended study vaccination
13. Individuals with history of any illness that, in the opinion of the investigator, might interfere with the results of the study or pose additional risk to the subject due to participation in the study
14. Individuals participating in any clinical trial with another investigational product 4 weeks prior to first study visit or intent to participate in another clinical study at any time during the conduct of this study.
15. Individuals who received any other vaccines within 4 weeks prior to enrollment in this study or who are planning to receive any vaccine within 4 weeks from the study vaccines
16. Individuals who have ever received blood, blood products and/or plasma derivatives or any parenteral immunoglobulin preparation in the past 12 weeks and for the full length of the study.
17. Individuals who are part of study personnel or close family members conducting this study.
18. BMI > 35 kg/m2.

4.3 Withdrawal of Subjects from Therapy or Assessment

The subject can withdraw consent for participation in the study at any time without penalty or loss of benefit to which the subject is otherwise entitled. The investigator can withdraw a subject if, in his or her clinical judgment, it is in the best interest of the subject or if the subject cannot comply with the protocol.

If a subject withdraws from the study, the reason for withdrawal should be documented in the subject's medical record and reported in the "Study Termination" CRF. If the withdrawal of a subject resulted from an Adverse Event, the "Adverse Events" and/or

“Prior and Concomitant Medications/Blood Products” CRFs, as applicable, should be completed.

For any subject who, despite the requirement for adequate contraception, becomes pregnant the site should maintain contact with them, complete a “Pregnancy Report” CRF as soon as possible, and obtain pregnancy outcome information for “Pregnancy Follow-up” CRF. The subject should be followed up after withdrawal and the reason for withdrawal (e.g. pregnancy) should be recorded in detail on the “Study Termination” CRF as well as on the subject’s medical records (see section 6.2.4 for further details).

Withdrawn subjects will not be replaced.

5.0 TREATMENT OF SUBJECTS

Each subject will receive a single 0.5 mL dose of the trivalent subunit non-adjuvanted influenza vaccine Optaflu.

All study related vaccines are to be kept in a secure location with appropriate storage conditions, temperature monitoring, and separate from other vaccines.

5.1 Investigational Vaccine

Test Vaccine: Optaflu

A single 0.5 mL dose of the study vaccine will be supplied in prefilled syringes and will be administered intramuscularly in the deltoid muscle of (preferably) the non dominant arm.

A 0.5 mL dose of Optaflu contains purified viral envelope-glycoproteins neuraminidase (NA) and hemagglutinin (HA), including 15 µg of HA of each of the three strains (A/H1N1-like strain, A/H3N2-like strain, B-like strain), recommended for inclusion in the vaccine composition for the influenza season 2011/2012 in the Northern Hemisphere.

Table 5.1-1 Investigational Vaccine Composition

**Vaccine Components**	
**Active ingredients**	**Unit formula per 0.5 mL**
A/ (H1N1)-like virus antigen	≥ 15 μg
A/ (H3N2)-like virus antigen	≥ 15 μg
B/ -like virus antigen	≥ 15 μg
**Excipients**	
sodium chloride	8.00 mg
potassium chloride	0.20 mg
potassium dihydrogen phosphate	0.37 mg
disodium phosphate dihydrate	1.29 mg
magnesium chloride hexahydrate	0.10 mg
Water for injection	up to 0.5 mL
**Volume of Formulation**	0,5 mL
**Appearance**	Liquid
**Vaccine Presentation**	Pre-filled syringe containing 0.5mL

5.2 Control Vaccines

Control vaccine will not be used for this trial.

5.3 Concomitant Vaccines or Treatment

Concomitant vaccine will not be used for this trial.

5.4 Vaccines Preparation and Administration

The principal investigator or designee will be responsible for the administration of the vaccine to subjects enrolled into the study according to the procedures stipulated in this study protocol. All vaccines will be administered only by personnel who are qualified to perform that function under applicable local laws and regulations for the specific study site.

PRECAUTIONS TO BE OBSERVED IN ADMINISTERING STUDY VACCINE:

The vaccine must be **well shaken** and visually inspected before use. The vaccination site should be disinfected with a skin disinfectant (e.g., 70% alcohol). Before vaccination, the skin must be dry. **DO NOT inject intravascularly**.

Standard immunization practices should be observed and care should be taken to administer the injection intramuscularly. As with all injectable vaccines, appropriate medical treatment and supervision should be readily available in case of anaphylactic reactions following administration of the study vaccine. Epinephrine 1:1000 and diphenhydramine or equivalent should be available in case of any anaphylactic reactions.

Study vaccines should not be administered to individuals with known hypersensitivity to any component of the vaccines.

Vaccinations must not be administered to any subject with a clinically significant active infection (as assessed by the investigator) or measured body (preferably axillary) temperature ≥ 38°C within 3 days of the intended date of vaccination. If either of these is observed, vaccination should be postponed until the subject's temperature remains below 38.0°C for at least 3 days or the investigator feels that the subject's illness has stabilized, as appropriate."

5.5 Other Concomitant Treatment or Vaccines

During this trial, medications taken by the subject prior to enrollment will not be collected.

Concomitant medications include all prescription medications (including non-study vaccines) taken by/administered to the subject after enrollment into the study and must be documented on the "Concomitant Medications /Blood Products" eCRF:

Use of the following concomitant medications after enrollment may interfere with the interpretation of the study objectives or indicate an underlying condition resulting in a major protocol violation according to the medical judgment of the Novartis Vaccines and Diagnostics physician.

The following concomitant treatments are discouraged and, if used, might indicate an underlying condition which may result in a major protocol violation according to the medical judgment of the Novartis Vaccines physician (see exclusion criteria):

a. Any parenteral or oral corticosteroid

b. Other immunosuppressive agents

c. Blood or plasma derivatives including immunoglobulin

d. Non-study vaccines (with the exception of post-exposure vaccinations in a medical emergency, e.g., hepatitis, rabies, tetanus).

5.6 Vaccination Compliance

The investigator is responsible for adequate and accurate accounting of vaccine usage. The investigator or designee will administer the study vaccines only to individuals included in this study following the procedures set out in this study protocol. The date, dosage, and time of the vaccinations must be recorded. The investigator must track vaccines received, used and wasted and will retain all unused or expired products as described in section 3.3.3.

6.0 EFFICACY/IMMUNOGENICITY AND SAFETY ASSESSMENTS

6.1 Appropriateness of Measurements

The measures of immunogenicity used in this study are standard, i.e., widely used and generally recognized as reliable, accurate, and relevant (able to describe the quality and extent of the immune response) and are according to CHMP criteria in terms of seroprotection, seroconversion or significant increase and geometric mean ratio (CPMP/BWP/214/96).

The measures of safety used in this study are routine clinical procedures. They include a close vigilance for, and stringent reporting of, selected local and systemic reactions routinely monitored in vaccine clinical trials as indicators of reactogenicity.

6.1.1 Immunogenicity

Serum responses to the three antigens contained in the vaccine (A/H1N1, A/H3N2 and B) will be assayed by HI assay. Antibodies maybe additionally quantified using the SRH test for confirmation purposes.(CPMP/BWP/214/96).

6.1.2 Methods, Criteria and Timing for Assessing and Recording Efficacy/Immunogenicity Parameters

Blood samples will be obtained prior to and 21 days (study day 22, window: -1+5 days) after vaccination.

HI and/or SRH antibody titers measured at Day 22 (21 days after 1st study vaccination) is the adequate timing to measure the antibody response of subjects. These titres will be compared against baseline antibody titers (Day 1, prior to vaccination) and will be used to evaluate immunogenicity.

Please see Section 7.5 for additional information regarding the assessment of immunogenicity parameters.

6.2 Safety Parameters

A brief medical history will be obtained and physical examination performed for each subject entered into the study.

Local and systemic reactions and other adverse events will be collected throughout the study, as detailed in sections 6.2.1 to 6.2.5.

6.2.1 Local and Systemic Reactions[1]

The occurrence of selected indicators of reactogenicity (listed below), which by definition, can only occur up to 3 days post vaccination, will be recorded on the "Local and Systemic Reactions" rather than the "Adverse Events" eCRF.

Local Reactions:

Ecchymosis, erythema, induration, swelling, pain

Systemic Reactions:

Chills/shivering, malaise, myalgia, arthralgia, headache, sweating, fatigue, fever (e.g. body temperature ≥ 38°C)

6.2.2 Adverse Events[2]

An adverse event (AE) is defined as any untoward medical occurrence in a subject or clinical investigation subject administered a pharmaceutical product at any dose that does not necessarily have to have a causal relationship with this treatment. An AE can, therefore be any unfavorable and unintended sign (including an abnormal laboratory finding, for example), symptom, or disease temporally associated with the use of an investigational product, whether or not considered related to the investigational product. This definition includes intercurrent illnesses or injuries and exacerbation of pre-existing conditions.

An unexpected adverse event is one that is not listed in the current Summary of Product Characteristics or the Investigator's Brochure or an event that is by nature more specific or more severe than a listed event.

All AEs will be monitored until resolution or, if the AE becomes chronic, a cause identified. If an AE is unresolved at the conclusion of the study, a clinical assessment

[1] Local and systemic reactogenicity is referred to as "solicited adverse events" to differentiate those events that were solicited on a checklist from other adverse events collected during the trial.
[2] Adverse Events collected in this study and that are not solicited on a checklist may also be referred to as "unsolicited Adverse Event" in the Clinical Study Report.

will be made by the investigator and medical monitor whether continued follow-up of the AE is warranted.

The severity of events reported on the "Adverse Events" CRF will be determined by the investigator as:

Mild:	transient with no limitation in normal daily activity.
Moderate:	some limitation in normal daily activity.
Severe:	unable to perform normal daily activity.

The relationship of the study treatment to an AE will be determined by the investigator based on the following definitions:

1. Not Related

The AE is not related if exposure to the investigational vaccine has not occurred, **or** the occurrence of the AE is not reasonably related in time, **or** the AE is considered unlikely to be related to use of the investigational vaccine, i.e. there are no facts (evidence) or arguments to suggest a causal relationship.

2. Possibly Related

The administration of the investigational vaccine and AE are considered reasonably related in time **and** the AE could be explained by causes other than exposure to the investigational vaccine.

3. Probably Related

Exposure to the investigational vaccine and AE are reasonably related in time **and** the investigational vaccine is more likely than other causes to be responsible for the AE, **or** is the most likely cause of the AE.

The relationship of the study treatment to an AE will be determined by the investigator.

6.2.3 Serious Adverse Events

A serious adverse event (SAE) is defined as any untoward medical occurrence that at any dose:

- Results in death
- Is life-threatening (i.e., the subject was, in the opinion of the investigator, at immediate risk of death from the event as it occurred); it does not refer to an event which hypothetically might have caused death if it were more severe.
- Requires or prolongs subject's hospitalization

- Results in persistent or significant disability/incapacity (i.e., the event causes a substantial disruption of a person's ability to conduct normal life functions)
- Results in a congenital anomaly/birth defect
- Is an important and significant medical event that may not be immediately life threatening or resulting in death or hospitalization but, based upon appropriate medical judgment, may jeopardize the subject or may require intervention to prevent one of the other outcomes listed above.

Adverse events which do not fall into these categories are defined as **non-serious**.

It should be noted that a severe adverse event need not be serious in nature and that a serious adverse event need not, by definition, be severe.

In addition, a pre-existing event or condition that results in hospitalization should be recorded on the Medical History CRF. If the onset of an event occurred before the subject entered the trial (e.g., any pre-planned hospitalization for conditions like cosmetic treatments or for non-emergency routine visits for a pre-existing condition), the hospitalization would not lead to an AE being classified as serious unless, in the view of the investigator, hospitalization was prolonged as a result of participation in the clinical trial or was necessary due to a worsening of the pre-existing condition.

6.2.4 Pregnancies

To ensure subjects' safety, each pregnancy in a subject on study vaccine must be reported to Novartis Vaccines and Diagnostics within 24 hours of learning of its occurrence. The pregnancy should be followed up to determine outcome, including spontaneous or voluntary termination, details of the birth, and the presence or absence of any birth defects, congenital abnormalities, or maternal and/or newborn complications.

Pregnancy should be recorded on a Clinical Trial Pregnancy Form and reported by the investigator to the local Novartis Clinical Safety & Epidemiology Department. Pregnancy follow-up should be recorded on the same form and should include an assessment of the possible relationship to the Novartis Vaccines and Diagnostics study vaccine of any pregnancy outcome. Any SAE experienced during pregnancy must be reported on the SAE Report Form.

6.2.5 Methods and Timing for Assessing and Recording Safety Parameters

All study subjects will be observed for approximately 30 minutes after the study vaccination for evidence of immediate reactions in general and in particular for symptoms of allergic phenomena (such as rashes, itching, or other allergic manifestations). Each subject, or where applicable, the subjects' parents or legally acceptable representative(s) will be instructed to complete a diary card for 3 days following the day of administration, to describe local and systemic reactions. If a local and systemic reaction continues

beyond 3 days after a vaccination, it will also be recorded on the “Adverse Events” eCRF. If the subject recovers on the last day, then this fact will be recorded on the “Comments” eCRF. All adverse events will be collected during Days 1 to 4. All adverse events necessitating a physician’s visit or consultation and/or leading to premature study discontinuation and all serious adverse events will be collected throughout the trial

All adverse events, regardless of severity, will be monitored by the investigator until resolution. All subjects experiencing adverse events - whether considered associated with the use of the study vaccine or not - must be monitored until symptoms subside and any abnormal laboratory values have returned to baseline, or until there is a satisfactory explanation for the changes observed, or until death, in which case a full pathologist’s report should be supplied, if possible. All findings must be reported on an “Adverse Events” eCRF and on the “Vaccine Serious Adverse Event” form, if necessary, which is part of the investigator’s study file. All findings in subjects experiencing adverse events must be reported also in the subject's medical records.

All SAEs which occur during the course of the trial, whether considered to be associated with the study vaccination or not, must be reported **within 24 hours** or at the latest on the following working day by telephone or fax to Sponsor or representative.

The “Vaccine Serious Adverse Event” form is to be completed for all SAEs and faxed to the Sponsor or representative. The original is retained by the investigator. The event is also documented on the “Adverse Events” eCRF. After receipt of the initial report, Sponsor or representative will review the information and contact the investigator if it is necessary to obtain further information for assessment of the event. Any medication or other therapeutic measures used to treat the event will be recorded on the appropriate eCRF(s) in addition to the outcome of the AE. Any serious adverse reaction must be reported to the EC in a timely manner. Adequate documentation will be provided to the sponsor showing that the EC has been properly notified. The sponsor must also comply with the applicable regulatory requirement(s) related to the reporting of unexpected serious and non-serious adverse vaccine reactions to the regulatory authority(ies) and the IRB/IEC.

If required, a follow-up report including all new information obtained on the serious adverse event must be prepared and sent to the Sponsor , or it will be collected by a representative of the sponsor. The report should be marked “Follow-up report.”

The investigator will submit, on request, copies of all these reports to the EC and other relevant authorities.

<u>Post-Study Events</u>

Any AE occurring at any time outside the observation period or after the end of the study and considered to be caused by the study vaccine - and therefore a possible adverse reaction - must be reported.

6.3 Data Monitoring Committee

There is no involvement of a Data Monitoring Committee during study conduct.

7.0 STATISTICAL PLAN

7.1 Statistical Hypothesis

There is no statistical null hypothesis associated with the immunogenicity objective.

7.2 Sample Size and Power Considerations

This study is in compliance with the sample size requirements of the current CHMP guideline on harmonization of requirements for influenza vaccines (CPMP/BWP/214/96).

7.3 Population for Analysis

Baseline is defined as the time of vaccination (Day 1).

Definition of populations to be analyzed:

(a) All Enrolled Set

- all subjects who:
  - have been enrolled and randomized into the study

(b) Exposed Set

- all subjects in the enrolled population who receive a study vaccination

(c) Full Analysis Set/Modified Intention-to-treat (MITT) population, Immunogenicity

- all subjects in the enrolled population who:
  - actually receive a study vaccination, and
  - provide evaluable serum samples before and after vaccination

(d) Per Protocol Set/ Immunogenicity

- all subjects in the Immunogenicity FAS who:
  - correctly receive the vaccine,
  - provide evaluable serum samples before and after vaccination
  - have no major protocol violation

A major deviation is defined as a protocol deviation that is considered to have a significant impact on the immunogenicity result of the subject.

(e) Safety Set

- all subjects in the Exposed Set who:
  - provide post vaccination safety data

Missing values will be left out in the appropriate analyses because they are regarded as non-informative. Data entry, data quality assurance, and statistical analyses will be conducted by the Biostatistics and Clinical Data Management (BCDM) group of Novartis Vaccines. All statistical analyses will be performed using SAS® version 9.1 or higher (SAS Institute, Cary, NC).

7.4 Analysis of Demographic and Baseline Characteristics

Summary statistics (mean, standard deviation, median, minimum and maximum) for age, height, weight and BMI at enrollment will be calculated. Distributions of subjects by sex, ethnic origin, year of last influenza vaccination and previous administration of influenza vaccine (yes/no) will be summarized.

Demography tables will be presented for the All Enrolled Set and the Per Protocol Set. Detailed subject listings will be located in the appendices (cf. Section 10

7.5 Analysis of Efficacy/Immunogenicity Endpoints

7.5.1 Description of Response Variables

In this study the titer as determined by HI assay test, it is defined as the reciprocal value of the dilution, e.g., a dilution of 1:40 represents a titre of 40. The detection limit of the HI assay is 10. All sera will be tested in duplicate, if an individual result is below detection limit it will be set to 5 in the laboratory. The geometric mean value of the duplicate test values will be received from the lab and used for analysis.

Regarding the titer as determined by the SRH assay, the diameter of the hemolytic zones observed including the well is provided by the laboratory. Zone areas (including the inner well) are calculated via the circle area function. The inner well has a diameter of 2.256mm thus the area of the inner well is $4mm^2$. Repeated measurements on the same sample are aggregated via the geometric mean of the respective areas.

Blood samples for the antibody assays will be drawn at the vaccination visit (Day 1) and 21 days after vaccination (Day 22, window: -1/+5).

7.5.2 Statistical Methods for Efficacy/Immunogenicity Variables

Immunogenicity

Immunogenicity analyses will be based on the PPS. All immunogenicity analysis tables will be done also for the FAS, if the PPS and FAS differ by more than 10% in the number of subjects included.

The measures of immunogenicity **for each antigen** are:

- the geometric mean area/titer (GMA/GMT) at Day 1 and at Day 22
- the Day 22/Day 1 geometric mean ratio (GMR)
- the percentage of subjects achieving seroconversion or significant increase in antibody titer
- the percentage of subjects achieving an SRH area ≥ 25 mm^2 or an HI titer ≥ 40 at Day 1 and at Day 22

where

In SRH tests, seroconversion or significant increase in antibody titer corresponds to:
negative prevaccination serum / postvaccination serum area ≥ 25 mm^2
at least a 50% increase in area from positive prevaccination serum
In HI tests, seroconversion or significant increase in antibody titer corresponds to:
negative prevaccination serum / postvaccination serum titer ≥ 40
at least a four-fold increase in titer from positive prevaccination serum

The following serological assessments will be considered for each strain in non-elderly adult subjects, aged between 18 and 60, and at least one of the assessments should meet the indicated requirements:

- The proportion of subjects achieving seroconversion or significant increase in HI titer or SRH area > 40%
- Mean geometric increase > 2.5
- The proportion of subjects achieving an HI titer ≥ 40 or SRH area ≥ 25 mm^2 should be > 70%

The following serological assessments will be considered for each strain in elderly subjects, aged 61 years and over, and at least one of the assessments should meet the indicated requirements:

- Proportion of seroconversion or significant increase in HI titer or SRH area > 30%

- Mean geometric increase > 2.0
- The proportion of subjects achieving an HI titer ≥ 40 or SRH area ≥ 25 mm^2 should be > 60%

Generally, all confidence intervals will be regarded as descriptive, and therefore, no adjustment to account for multiplicity will be made.

7.6 Analysis of Safety (Endpoints) and Tolerability

All subjects who receive the vaccination and provide some safety data will be considered evaluable for the safety analyses. The safety of the study vaccines will be assessed in terms of number of subjects exposed to study vaccines with reported local and systemic reactions, as well as the number of all subjects with reported SAEs and/or AEs (as specified for each time period) per age group. The safety analyses also will include data from the physical examination and any reactions or AEs observed by study personnel following vaccination. All SAEs and AEs (including onset of chronic illness) will be judged by the Investigator as either, probably related, possibly related, or not related to vaccine and will be tabulated. All SAEs and AEs resulting in withdrawal from the study will be summarized.

7.6.1 Analysis of Extent of Exposure

As vaccination consists of only one fixed dose, related information is presented as standard listing of subject vaccination data.

7.6.2 Analysis of Local and Systemic Reactions

Frequencies and percentages of subjects experiencing each reaction will be presented for each symptom severity. Summary tables showing the occurrence of any local or systemic reaction overall and at each time point will also be presented.

Post-vaccination reactions reported from day 1 to day 4 will be summarized by maximal severity and by vaccine group. The severity of local reactions, including injection-site ecchymosis, erythema, induration, and swelling will be categorized as none, 1 to ≤10 mm, 11 to ≤25 mm, 26 to ≤50mm, 51 to ≤100 mm and >100 mm (severe local reactions).

The severity of pain and systemic reactions (chills/shivering, malaise, myalgia, arthralgia headache, sweating and fatigue) occurring up to 4 days after each vaccination will be categorized as none, mild (transient with no limitation in normal daily activity), moderate (some limitation in normal daily activity), and severe (unable to perform normal daily activity).

Each local and systemic reaction will also be categorized as none vs. any. Body (preferably axillary) temperature will be categorized as <38°C (no fever), 38-38.9°C, 39-39.9°C and ≥40°C (severe).

If comparisons will be made for the local and systemic reaction safety variables, differences among the vaccine groups after each scheduled immunization and after any immunization with respect to all variables (including fever) will be analyzed by using Pearson's chi-square test, or Fisher's Exact test where appropriate.

7.6.3 Analysis of Other Adverse Events

All the adverse events occurring during the study, judged either as related to vaccination or not by the investigator, will be recorded as specified in section 6.2.5. The original verbatim terms used by investigators to identify adverse events in the eCRFs will be mapped to preferred terms using the MedDRA dictionary. The adverse events will then be grouped by MedDRA preferred terms into frequency tables according to system organ class. All reported adverse events, as well as adverse events judged by the investigator as at least possibly related to study vaccine, will be summarized according to system organ class and preferred term within system organ class. These summaries will be presented by age group. When an adverse event occurs more than once for a subject, the maximal severity and strongest relationship to the vaccine group will be counted. Additionally, three separate summaries will be produced: (i) serious adverse events, (ii) adverse events that are possibly or probably related to vaccine, and (iii) adverse events that are unrelated to vaccine. Data listings of all adverse events will be provided by subject. In addition, a listing of subjects withdrawn from the study because of an adverse event will be presented, as well as a listing of adverse events leading to hospitalization.

7.7 Planned Interim Analysis

No interim analysis of data from this trial is planned. Should it later become necessary, the analysis will be governed by the procedures specified in the Novartis Vaccines SOP entitled "Interim Analysis".

8.0 STUDY MONITORING, AUDITING AND DOCUMENTATION

Study monitoring and auditing will be performed in accordance with the sponsor's standard operating procedures and applicable regulatory requirements (e.g., FDA, EMEA, ICH and GCP guidelines).

Investigators and/or their study staff will be trained on the study protocol and all applicable study procedures prior to subject enrollment. CRFs supplied by the sponsor must be completed for each enrolled subject. The data entries as well as study related documents will be checked by the sponsor and/or trained delegates of the sponsor.

8.1 Study Monitoring

Study progress will be monitored by Novartis Vaccines and Diagnostics or its representative (e.g. a contract research organization) as frequently as necessary to ensure the rights and well-being of study subjects are protected, to verify adequate, accurate and

complete data collection, protocol compliance and to determine that the study is being conducted in conformance with applicable regulatory requirements. Arrangements for monitoring visits will be made in advance in accordance with the monitoring plan, except in case of emergency.

8.2 Source Data Verification

Data recorded on the eCRF will be verified by checking the CRF entries against source documents (i.e., all original records, laboratory reports, medical records, subjects diaries) in order to ensure data completeness and accuracy as required by study protocol. The investigator and/or site staff must make CRFs and source documents of subjects enrolled in this study available for inspection by Novartis Vaccines and Diagnostics or its representative at the time of each monitoring visit.

At a minimum, source documentation must be available to substantiate subject identification, eligibility and participation, proper informed consent procedures, dates of visits, adherence to protocol procedures, adequate reporting and follow-up of adverse events, administration of concomitant medication, study vaccine receipt/dispensing/return records, study vaccine administration information, and date of completion and reason. Specific items required as source documents will be reviewed with the investigator before the study

The source documents must also be available for inspection, verification and copying, as required by regulations, by officials of the regulatory health authorities (e.g. FDA, EMEA and others) and/or ECs/IRBs. The investigator and study site staff must comply with applicable privacy, data protection and medical confidentiality laws for use and disclosure of information related to the study and enrolled subjects.

The subject or the subject's parents or legally acceptable representative(s) must also allow access to the subject's medical records. Each subject, or the subject's parent(s) or legally acceptable representative(s), should be informed of this prior to the start of the study.

9.0 DATA MANAGEMENT

An electronic data capture (EDC) system (e.g., Inform™) will be used to expedite the entry of data. The investigator will enter data into the web enable EDC system in a timely manner; the data will be stored in Novartis Vaccines and Diagnostics' clinical database management system. eCRF data will be reviewed routinely by Novartis Vaccines and Diagnostics BCDM Group and Novartis Vaccines and Diagnostics clinical monitors.

All serology data analyzed by Clinical Serology, Novartis Vaccines and Diagnostics, Marburg will be entered into the Seroad database by Novartis Vaccines's Clinical Serology Laboratory, Marburg. All results will be checked in the laboratory for validity and completeness.

Electronic Data Transfer (EDT) is one method being used by Novartis Vaccines and Diagnostics for collecting laboratory data. The Molecular Epidemiology laboratory of the Department of Physiopathology, Experimental Medicine and Public Health, University of Siena will send data as electronic files by a secured method (e.g., via diskette, CD, as an encrypted file attachment on electronic mail, or as a direct transfer into a specified server directory) to Novartis Vaccines's and Diagnostics BCDM department. The data file is pre-processed and loaded by the BCDM Lab Manager into the study database. The laboratory will submit a results' file containing the tests and the results as specified in the protocol.

9.1 Data Handling Procedures

Coding will be performed using the following dictionaries:

Adverse Events:	MedDRA
Concomitant illness:	ICD-9
Concomitant and intercurrent therapy:	WHO Drug Dictionary

9.2 Documentation of Study Findings

The investigator must review and electronically sign the eCRFs to verify their accuracy. Correction to data on eCRFs will be tracked via an audit trail within InForm™, web based electronic data capture system. Each correction will be identified by the person making the change and will include time, date, and reason for change. If corrections are made to a previously and electronically signed CRF, the investigator, he or she must confirm and endorse the changes.

As part of the conduct of the trial, Novartis Vaccines and Diagnostics may have questions about the Case Report Form data after the site has entered the data. These questions will be raised within InForm™. The Investigator will provide follow-up clarification and/or resolution of data issues raised by the monitor or the data manager.

An explanation must be provided and documented by the investigator for all missing data.

9.3 Data Protection

Novartis Vaccines and Diagnostics respects the subjects' rights to privacy and will ensure the confidentiality of their medical information in accordance with all applicable laws and regulations.

The sponsor as Data Controller according to the European Directive on the protection of individuals with regard to the processing of personal data and on the free movement of

such data [95/46/EC] confirms herewith compliance to Directive 95/46/EC in all stages of Data Management.

10.0 RECORD RETENTION

Investigators must retain all study records required by Novartis Vaccines and Diagnostics and by the applicable regulations in a secure and safe facility. The investigator must consult a Novartis Vaccines and Diagnostics representative before disposal of any study records, and must notify the sponsor of any change in the location, disposition, or custody of the study files. Essential documents must be retained until at least 2 years after the last approval of a marketing application in an ICH region and until there are no pending or contemplated marketing applications in an ICH region or at least 2 years have elapsed since the formal discontinuation of clinical development of the investigational product. "Essential documents" are defined as documents that individually and collectively permit evaluation of the conduct of a trial and the quality of the data produced. These documents should be retained for a longer period however, if required by the applicable regulatory requirements or by an agreement with the sponsor *or* The Committee for Human Medicinal Products fro Human Use (CHMP) requires retention for the maximum period of time permitted by the institution, but not less than 15 years. (ICH E6, 4.9.5) It is the responsibility of the sponsor to inform the investigator/institution as to when these documents no longer need to be retained. (ICH E6, 5.5.12).
These principles of record retention will also be applied to the storage of laboratory samples, provided that the integrity of the stored sample permits testing.

11.0 USE OF INFORMATION AND PUBLICATION

Novartis Vaccines and Diagnostics assures that the key design elements of this protocol will be posted in a publicly accessible database such as clinicaltrials.gov.

Novartis Vaccines and Diagnostics also assures that key results of this clinical trial will be posted in a publicly accessible database within one year from the last subject's last study visit (LSLV).

12.0 ETHICS

12.1 Regulatory and Ethical Compliance

This clinical study was designed and shall be implemented and reported in accordance with the ICH Harmonized Tripartite Guidelines for Good Clinical Practice, with applicable local regulations (including European Directive 2001/20/EC, US Code of Federal Regulations Title 21, and Japanese Ministry of Health, Labor, and Welfare), and with the ethical principles laid down in the Declaration of Helsinki.

12.2 Informed Consent Procedures

Eligible subjects may only be included in the study after providing written (witnessed, where required by law or regulation), IRB/IEC/REB-approved informed consent, or, if incapable of doing so, after such consent has been provided by a legally acceptable representative of the subject. In cases where consent is given by the subject's representative, the subject should be informed about the study to the extent possible given his/her understanding. If the subject is capable of doing so, he/she should indicate assent by personally signing and dating the written informed consent document or a separate assent form. Informed consent must be obtained before conducting any study-specific procedures (i.e. all of the procedures described in the protocol). The process of obtaining informed consent should be documented in the subject source documents.

Novartis Vaccines and Diagnostics will provide to investigators a separate document with a proposed informed consent form that complies with the ICH GCP guideline and regulatory requirements and is considered appropriate for this study. Any changes to the proposed consent form suggested by the investigator must be agreed to by Novartis Vaccines and Diagnostics before submission to the IRB/IEC/REB, and a copy of the approved version must be provided to the Novartis Vaccines and Diagnostics monitor after IRB/IEC/REB approval.

Women of child bearing potential should be informed that taking the study medication may involve unknown risks to the fetus if pregnancy were to occur during the study and agree that in order to participate in the study they must adhere to the contraception requirements indicated in the protocol for the duration of the study. If case of doubts on the ability of a subject to adhere to these requirements, that subject should not be allowed in the study

12.3 Responsibilities of the Investigator and IRB/IEC/REB

The protocol and the proposed informed consent form must be reviewed and approved by a properly constituted Institutional Review Board/Independent Ethics Committee/Research Ethics Board (IRB/IEC/REB) before study start. A signed and dated statement that the protocol and informed consent have been approved by the IRB/IEC/REB must be given to Novartis Vaccines and Diagnostics before study

initiation. Prior to study start, the investigator is required to sign a protocol signature page confirming his/her agreement to conduct the study in accordance with these documents and all of the instructions and procedures found in this protocol and to give access to all relevant data and records to Novartis Vaccines and Diagnostics monitors, auditors, Novartis Clinical Quality Assurance representatives, designated agents of Novartis Vaccines and Diagnostics, IRBs/IECs/REBs, and regulatory authorities as required. If an inspection of the clinical site is requested by a regulatory authority, the investigator must inform Novartis Vaccines and Diagnostics immediately that this request has been made.

12.4 Protocol Adherence

Investigators will apply due diligence to avoid protocol deviations. Under no circumstances should the investigator contact Novartis Vaccines and Diagnostics or its agents, if any, monitoring the trial to request approval of a protocol deviation, as no authorized deviations are permitted. If the investigator feels a change to the protocol would improve the conduct of the study this must be considered a protocol amendment, and unless such an amendment is agreed upon by Novartis Vaccines and Diagnostics and approved by the IRB/IEC/REB it cannot be implemented. All significant protocol deviations will be recorded and reported in the CSR.

12.5 Protocol Amendments

An amendment is a written description of change(s) to or formal clarification of a study protocol which may impact on the conduct of the clinical study, potential benefit of the clinical study, or may affect subject safety, including changes of study objectives, study design, subject population, sample sizes, study procedures, or significant administrative aspects. An administrative change of a study protocol is a minor correction or clarification that has no significant impact on the way the clinical study is to be conducted and no effect on subject safety (e.g., change of telephone number(s), logistical changes). Protocol amendments must be approved by Novartis Vaccines and Diagnostics, Health Authorities where required, and the IRB/IEC/REB. In cases when the amendment is required in order to protect the subject safety, the amendment can be implemented prior to IRB/IEC/REB approval. Notwithstanding the need for formal approval of a protocol amendment, the investigator is expected to take any immediate action required for the safety of any subject included in this study, even if this action represents a deviation from the protocol. In such cases, Novartis Vaccines and Diagnostics should be notified of this action and the IRB/IEC/REB at the study site should be informed within 10 working days.

13.0 REFERENCE LIST

1. Cox NJ, Subbarao K. Influenza. Lancet 1999; 354: 1277-82.

2. Simonsen L, Schonberger LB, Stroup DF, Arden NH, Cox NJ. Impact of influenza on mortality in the USA. In: Brown LE, Hampson AW, Webster RG, eds. Options for the control of influenza III: proceedings of the 3rd International Conference on Options for the Control of Influenza, Cairns, Australia, 4--9 May, 1996. Amsterdam, Holland: Elsevier Science; 1996; 26-33.

3. Cram P, Blitz SG, Monto A, Fendrik AM. Influenza. Cost of illness and considerations in the economic evaluation of new and emerging therapies. Pharmacoeconomics 2001; 19 (3): 223-30.

4. Fleming DM. The contribution of influenza to combined acute respiratory infections, hospital admissions and death in winter. Commun Dis Public Health 2000; 3: 32-8.

5. Noble GR. Epidemiological and clinical aspects of influenza. In: Beare AS, ed. Basic and applied influenza research. Boca Raton, Fla.: CRC Press; 1982; 11-50.

6. Mulloly JP, Barker WH. Impact of type A influenza on children: a retrospective study. Am J Public Health 1982; 72: 1008-16.

7. Hall CB. Respiratory syncytial virus. In: Feigin RD, Cherry JD, eds. Textbook of pediatric infectious diseases. 4th ed. Vol. 2. Philadelphia: W.B. Saunders; 1998; 2084-111.

8. Eickhoff TC, Sherman IL, Serfling RE. Observations on excess mortality associated with epidemic influenza. JAMA 1961; 176: 776-82.

9. Glezen WP, Decker M, Perrotta DM. Survey of underlying conditions of persons hospitalized with acute respiratory disease during influenza epidemics in Houston, 1978-1981. Am Rev Respir Dis 1987; 136: 550-5.

10. Neuzil KM, Reed GW, Mitchel EF, Simonsen L, Griffin MR. Impact of influenza on acute cardiopulmonary hospitalizations in pregnant women. Am J Epidemiol 1998; 148: 1094-102.

11. Barker WH. Excess pneumonia and influenza-associated hospitalization during influenza epidemics in the United States, 1970-78. Am J Public Health 1986; 76: 761-65.

12. Kendal AP. Epidemiologic implications of changes in the influenza virus genome. Am J Med 1987; 82 (6A): 4-14.

13. Manuguerra JC, Mosnier A on behalf of EISS (European Influenza Surveillance Scheme). Surveillance of influenza in Europe from October 1999 to February 2000. Eurosurveillance 2000; 5: 63-8.

14. Nichol KL, Lind A, Margolis KL, et al. Effectiveness of vaccination against influenza in healthy, working adults. N Engl J Med 1995; 333: 889-93.

15. Nichol KL, Wuorenma J, Von Sternberg T. Benefits of influenza vaccination for low-, intermediate-, and high-risk senior citizens. Arch Intern Med 1998; 158: 1769-76.

16. Bridges CB, Thompson WW, Meltzer MI, et al. Effectiveness and cost-benefit of influenza vaccination of healthy working adults: a randomized controlled trial. JAMA 2000; 284: 1655-63.

17. Patriarca PA, Weber JA, Parker RA, et al. Risk factors for outbreaks of influenza in nursing homes: a case-control study. Am J Epidemiol 1986: 124; 114-9.

18. Gross PA, Hermogenes AW, Sacks HS, Lau J, Levandowski RA. Efficacy of influenza vaccine in elderly persons: a meta-analysis and review of the literature. Ann Intern Med 1995; 123: 51-27.

19. Mullooly JP, Bennett MD, Hornbrook MC, et al. Influenza vaccination programs for elderly persons: cost-effectiveness in a health maintenance organization. Ann Intern Med 1994; 121: 947-52.

20. Clements DA, Langdon L, Bland C, Walter E. Influenza A vaccine decreases the incidence of otitis media in 6- to 30-month-old children in day care. Arch Pediatr Adolesc Med 1995; 149: 1113-7.

21. Centers for Disease Control and Prevention. Prevention and control of influenza. Recommendations of the Advisory Committee on Immunization Practices (ACIP). MMWR 2001; 50 (RR04): 1-46.

22. AD HOC WORKING PARTY ON BIOTECHNOLOGY/PHARMACY, Meeting on Influenza Vaccine, Commission of the European Countries, March 1992.

23. Committee for Medicinal Products for Human Use (CHMP). Note for Guidance on Harmonisation of Requirements for Influenza Vaccines. CPMP/BWP/214/96: 12 March 1997.

24. WHO. Cell culture as a substrate for the production of influenza vaccines: memorandum from a WHO meeting. *Bull World Health Organ* 1995; 73: 431-5

25. TAMBLYN SE, HINMAN AR. Pandemic planning: conclusions and recommendations. In: Hannoun C, Kendal AP, Klenk HD, Ruben FL, eds. Options for the control of influenza II. Amsterdam: Elsevier Science Publishers, 1993: 457-9
